# First principles and machine learning investigation of structural stability and optoelectronic behavior in A_2_GaAgF_6_ (A = Na, K, Rb, Cs) double perovskite solar cells

**DOI:** 10.1038/s41598-026-49631-8

**Published:** 2026-04-21

**Authors:** Asadul Islam Shimul, Karim Kriaa, Bipul Chandra Biswas, Chemseddine Maatki, Md. Azizur Rahman, Mekuria Tsegaye Alemu, Noureddine Elboughdiri

**Affiliations:** 1 Department of Electrical and Electronic Engineering, Gopalganj Science and Technology University, Gopalganj, 8105 Bangladesh; 2 College of Engineering, Imam Mohammad Ibn Saud Islamic University (IMSIU), 11432 Riyadh, Saudi Arabia; 3 Renewable Energy & Nanoelectronics Research Laboratory, Department of Electrical and Electronic Engineering, Begum Rokeya University, Rangpur, 5400 Bangladesh; 4 Department of Physics, College of Natural and Computational Science, Kebri Dehar University, P.O. Box 250, Kebri Dehar, Ethiopia; 5 Chemical Engineering Department, College of Engineering, University of Ha’il, P.O. Box 2440, Ha’il, 81441 Saudi Arabia

**Keywords:** A_2_GaAgF_6_, Machine learning, DFT, SCAPS-1D, Double perovskite solar cells, Engineering, Materials science, Physics

## Abstract

This research examines the structural, electronic, phonon, mechanical, and optical characteristics of alkali-based double perovskites A_2_GaAgF_6_ (where A = Na, K, Rb, and Cs) through Density Functional Theory (DFT) simulation. All compounds demonstrate both dynamic and mechanical stability, with direct band gaps between 1.5 and 2.7 eV, suggesting ductile behavior and anisotropy. To further evaluate device performance, One-dimensional Solar Cell Capacitance Simulator (SCAPS-1D) simulations were performed. A total of 196 device architectures were investigated by integrating seven electron transport layers (ETLs: C_60_, In_2_S_3_, CdS, ZnO, IGZO, SnS_2_, and MZO) with seven-hole transport layers (HTLs: CFTS, CuI, Sb_2_S_3_, PTAA, CuSbS_2_, MoO_3_, and MoTe_2_). Among these configurations, four structures were examined in detail. The Al/FTO/In_2_S_3_/Na_2_GaAgF_6_/PTAA/Ni device demonstrates highest power conversion efficiency (PCE) of 28.87%, accompanied by a short-circuit current density (J_SC_) of 25.55 mA/cm^2^, a fill factor (FF) of 89.92%, and an open-circuit voltage (V_OC_) of 1.256 V. Devices utilizing K_2_GaAgF_6_, Rb_2_GaAgF_6_, and Cs_2_GaAgF_6_ demonstrated PCEs of 14.89%, 12.38%, and 7.09% respectively. The effects of contact materials, band alignment, absorber thickness, defect density, doping concentration, and interface defect characteristics were analyzed systematically. To accelerate device optimization, machine learning based on the Random Forest algorithm was developed. Analysis of feature importance indicated that the absorber band gap and defect density are the primary factors influencing device efficiency. The model exhibited high predictive accuracy, with Root Mean Square Error (RMSE) of 0.177 and coefficient of determination (R^2^) of 0.972, indicating strong alignment with SCAPS-1D simulation results. This integrated framework provides a reliable and effective method for designing lead-free, sustainable, and high-performance double perovskite solar cells.

**Supplementary Information:**

The online version contains supplementary material available at 10.1038/s41598-026-49631-8.

## Introduction

Considering the growing significance of climate change on ecosystems, health, and economies, it is imperative to formulate long-term energy efficiency and sustainability policies. Perovskite-based materials have arisen as viable options owing to their adjustable bandgap, distinctive crystal structures, and superior optoelectronic performance^1–3^. Nonetheless, lead-based perovskites present significant toxicological and environmental hazards due to lead and volatile synthesis intermediates^4^. In response to these issues, international research has transitioned to lead-free alternatives that preserve excellent performance while mitigating detrimental impacts. Among these, lead-free double perovskites (LFDPs) are particularly appealing. These materials attain charge neutrality and structural stability by substituting divalent cations (B^2+^) with monovalent (B^1+^) and trivalent (B^3+^) cations, in accordance with the A_2_BB’X_6_ formula^5^. Double perovskites (DPs) often exhibit a cubic Fm3̅m (225) structure with lattice constants ranging from 8 to 12 Å^6^, providing enhanced mechanical strength, tunability, and resistance to heat and moisture. Their adaptable electrical characteristics provide precise regulation of charge transport and band structure, rendering them formidable candidates for sustainable energy and optoelectronic applications^7,8^. Recent research demonstrates the extensive adaptability of LFDPs in energy and optoelectronic applications. Shen et al.^9^ demonstrated that alkaline-earth-metal-based perovskite hydrides possess superior hydrogen storage capacity, whereas Lu et al.^10^ and Fang et al.^11^ established that the tunable lattice dynamics and efficient charge transport of LFDPs. Solid-state produced LFDP thermal barrier coatings have poor thermal conductivity attributed to phonon scattering caused by lattice distortion and cation disorder, hence providing good thermal resistance and mechanical stability for aerospace applications.

Extensive theoretical and experimental endeavors have focused on identifying and optimizing DPs comprising Li, Na, K, Rb, Cs, Ga, Ag, Au, Tl, As, Al, In, and Mn cations in conjunction with halides such as F, Cl, Br, and I^12–14^. Research on compounds such as Na_2_AlAgF_6_, Li_2_AgAsBr_6_, K_2_ScInI_6_, Rb_2_MgGeI_6_, and Cs_2_NaIrCl_6_ has demonstrated favorable optical properties; yet, the majority exhibit comparatively broad band gaps, constraining their potential for photovoltaic (PV) commercialization^15–17^. Owing to fabrication difficulties, a limited quantity of research has empirically investigated the comprehensive PV performance of these materials. As a result, researchers are progressively utilizing SCAPS-1D simulations to forecast PV performance and enhance device layouts. Among those, Uddin et al.^18^ employed DFT to investigate the lead-free Cs_2_AgBiBr_6_ perovskite, uncovering a bandgap of 1.654 eV and robust optical absorption suitable for solar cells. The FTO/AZnO/Cs_2_AgBiBr_6_/CNTS/Au configuration, with CNTS as the HTL, attained an efficiency of 23.5%, indicating stable and non-toxic PV potential. Deswal et al.^19^ utilized SCAPS-1D to enhance Cs_2_AgInBr_6_ double perovskite solar cells (DPSCs), demonstrating that a 600 nm absorber achieves 26.9% efficiency, underscoring its potential as a high-performing, lead-free material. Chargui et al.^20^ investigated Cs_2_ScAgX_6_ (X = Br, I), determining bandgaps of 1.8 and 1.55 eV, exhibiting robust absorption conducive for tandem cells. A 490 nm top cell combined with a CIGS bottom cell attained a 28.88% efficiency. Khan et al.^13^ examine LFDPs X_2_MgGeI_6_ (X = Rb, Cs) utilizing WIEN2k and SCAPS-1D, uncovering indirect bandgaps of 1.5 and 1.495 eV, along with remarkable stability and optical characteristics. Both compounds exhibit significant potential for solar cells and photocatalytic water splitting, attaining remarkable power conversion efficiencies of 30–32%. The selection of cations and halide anions (F, Cl) significantly affects lattice distortion, electrical configuration, and band alignment in perovskites. More substantial halides, such as florin, enlarge the lattice, so adjust the bandgap and symmetry. Comprehending the structural-electronic interactions in systems that integrate alkali metals (Li, Na, Rb, Cs) with trivalent metals like Ag is essential for the development of efficient, lead-free PV materials. Due to their restricted research, Ag-based perovskites necessitate further investigation into their electrical and optical characteristics^21^.

The structural, electrical, and optical properties of alkali-based double perovskites A_2_GaAgF_6_ (A = Na, K, Rb, Cs) were examined using DFT to evaluate their viability as lead-free photovoltaic absorbers. The preference for fluoride-based systems instead of the more extensively researched iodide or bromide perovskites is due to their enhanced chemical stability, stronger metal-fluorine interactions, and less ionic migration, all of which are essential for improving long-term device reliability^22^. In contrast to iodide perovskites like Cs_2_AgBiI_6_, which demonstrate favorable optical tunability yet experience degradation due to humidity and temperature stress, the Ga-Ag-F framework offers a more robust lattice and significantly reduces defect-mediated recombination^23^. The optimized A_2_GaAgF_6_ compounds exhibit cubic Fm-3 m symmetry, aligning with previously documented fluoride counterparts such as Na_2_AlAgF_6_ and Cs_2_AgBiF_6_^15,24^. The lattice constants consistently increase from Na to Cs, demonstrating expected structural flexibility, while investigations of elastic constants and phonon dispersion verify that all compositions exhibit both mechanical and dynamical stability. The calculated direct band gaps fall within the ideal range for visible-light absorption, consistent with previous findings on Ag-Ga-F-based perovskites. Moreover, the elevated ionicity and minimal defect density of the F-Ag-Ga lattice enhances carrier lifetimes and diminish non-radiative losses. These collectively indicate that fluoride-based A_2_GaAgF_6_ compounds possess structural robustness, chemical stability, and advantageous optoelectronic properties, positioning them as viable, sustainable alternatives to traditional lead-halide perovskites for next-generation solar energy applications.

To further investigate the PV efficacy of A_2_GaAgF_6_ (A = Na, K, Rb, Cs) materials as absorber layers, SCAPS-1D simulations were implemented. A total of 196 device combinations were examined by integrating seven ETLs with seven HTLs. Four optimized device architectures of the general configuration front contact/FTO/ETL/A_2_GaAgF_6_/HTL/back contact were thoroughly examined. The results of the simulation suggested that Na_2_GaAgF_6_-based devices demonstrated enhanced band alignment, carrier mobility, and charge extraction efficiency, culminating in the highest PCE of all compositions. Additionally, parametric analysis of defect density, absorber thickness, band offset, doping concentration, and quantum efficiency (QE) highlighted their significant impact on device performance. The outcomes suggest that Na_2_GaAgF_6_ is a viable, eco-friendly, and structurally robust absorber for advanced DPSCs.

## Computational methodology

Density Functional Theory (DFT) simulations were conducted utilizing the CASTEP algorithm to examine the structural, electrical, and mechanical properties of A_2_GaAgF_6_ (where A = Na, K, Rb, and Cs). The exchange–correlation interactions were characterized using the Perdew–Burke–Ernzerhof (PBE) functional within the Generalized Gradient Approximation (GGA), offering a dependable equilibrium between computational efficiency and precision for halide perovskite systems^25^. The interactions between valence electrons and ionic cores were addressed utilizing Vanderbilt-type ultrasoft pseudopotentials. A plane-wave basis set with a kinetic energy cutoff of 700 eV was utilized to guarantee precise total energy convergence. Brillouin zone integrations were performed with a Monkhorst–Pack k-point mesh of 9 × 9 × 9, which was verified to yield well-converged results for total energy and electronic characteristics. Structural optimization was deemed converged when the overall energy variation between consecutive ionic steps fell below 1 × 10^–6^ eV per unit cell, and the residual Hellmann–Feynman forces on each atom were under 0.002 eV/Å^26^. The Broyden–Fletcher–Goldfarb–Shanno (BFGS) minimization algorithm was employed to achieve the optimum ground-state configurations^27^. The elastic stiffness constants (C_ij_) and associated mechanical parameters were ascertained via the stress–strain method as executed in CASTEP^28^. Phonon dispersion simulations were performed to verify the dynamical stability of the improved structures. The influence of spin–orbit coupling (SOC) on the electronic characteristics was assessed, given the presence of relatively heavy materials like Ag and Cs^16,29^. Test computations, including SOC, revealed just a slight alteration in the band gap values without affecting the main properties of the band structure or the nature of the bandgap. Consequently, SOC was excluded from the final computations due to its insignificant impact and substantial increase in computing expense. Alongside first-principles calculations, SCAPS-1D simulations were conducted to assess the photovoltaic performance of the proposed materials. The SCAPS-1D simulator relies on the numerical resolution of three essential semiconductor equations: Poisson’s equation and the continuity equations for electrons and holes, which delineate the spatial variation of electrostatic potential and charge carrier distributions within the device^30,31^. The coupled differential equations were solved self-consistently to examine the optoelectronic properties of the engineered solar cell topologies. Poisson’s equation can be articulated as Eq. (1):

1 $$\frac{\partial }{\partial x}\left(\varepsilon (x)\frac{\partial \Psi }{\partial x}\right)= -\frac{q}{{\varepsilon }_{o}}\left[{N}_{D}^{+}-{N}_{A}^{-}+p-n\right]-\frac{{\rho }_{def}\left(n, p\right)}{{\varepsilon }_{o}}$$

The electrostatic potential is denoted by $\Psi$, the dielectric constant by ε, the elementary electronic charge by q, and the defect charge density by ${\rho }_{def}$. Equations (2) and (3), which denote the continuity equations for electrons and holes, articulate the conservation of free charge carriers within the device.

2 $$\frac{\partial n}{\partial t}+\frac{\partial {J}_{n}}{\partial n}=G-{U}_{n}\left(n, p\right)$$

3 $$\frac{\partial p}{\partial t}+\frac{\partial {J}_{p}}{\partial n}=G-{U}_{p}\left(n, p\right)$$

In these equations, J_n_ and J_p_ denote the electron and hole current densities; ${U}_{n, p}$ signify the net recombination rates; and G represents the carrier generation rate. Furthermore, A machine learning-based Random Forest technique was subsequently employed on the Na_2_GaAgF_6_-based PSC utilizing a simulator-generated dataset comprising 19,200 data entries. A total of 15,360 samples (80%) were utilized for model training, and the remaining 3,840 samples (20%) were allocated for testing and assessing the model’s predicted accuracy. The model’s effectiveness was assessed using conventional statistical metrics: MSE, MAE, RMSE, and R^2^^32^. Additionally, correlation heatmap and SHAP analysis were performed to clarify the relative significance of several input parameters on the performance of the PSC.

## Results and discussion

### Structural attributes

**Fig. 1:**
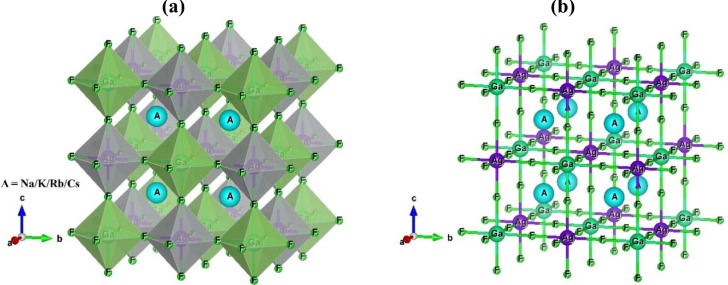
Graphical visualization of the A_2_GaAgF_6_ (A = Na, K, Rb, and Cs) double perovskites: (**a**) polyhedral representation and (**b**) ball-and-stick model demonstrating the atomic configuration.

The structural assessment of crystalline materials is crucial for comprehending their atomic arrangements, which directly determine the materials’ chemical composition and physicochemical properties. The atomic configuration of the A_2_GaAgF_6_ (A = Na, K, Rb, Cs) double perovskites were meticulously analyzed to ascertain their geometric and structural characteristics. These elements exhibit cubic arrangement with space group Fm-3 m (No. 225), signifying a face-centered cubic (FCC) architecture typical of the elpasolite-type double perovskites family^33^. Within this framework, two separate octahedral coordination environments are identified. The Ga^3+^ cation occupies one octahedral site, coordinated by six F^-^ anions to create a GaF_6_ octahedron, while the Ag^+^ cation occupies an alternate site, making a corresponding AgF_6_ octahedron. The two octahedral units alternate in a rock-salt arrangement, sharing fluorine corners, so ensuring the structural integrity and periodicity of the lattice. The alkali-metal cations (Na⁺, K⁺, Rb⁺, Cs⁺) reside interstitially in the larger A-sites between the octahedra, ensuring charge neutrality and bolstering the structural integrity of the crystal lattice. The precise atomic coordinates within the cubic unit cell are as follows: the A-site cation is located at (0.75, 0.25, 0.25), the Ga atom at (0, 0, 0), Ag at (0.5, 0, 0), and F at (0.75, 0, 0), as shown in Fig. 1a,b. This periodic structure illustrates the consistency and three-dimensional interconnectivity of the atomic framework in the perovskite lattice, essential for comprehending the compound’s electrical and thermodynamic properties.

**Fig. 2:**
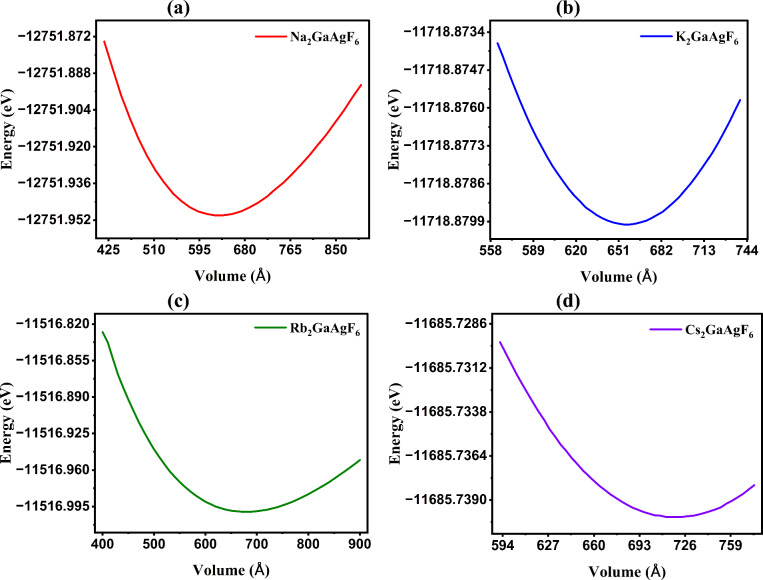
Ground-state energy as a function of unit-cell volume for (**a**) Na_2_GaAgF_6_, (**b**) K_2_GaAgF_6_, (**c**) Rb_2_GaAgF_6_, and (**d**) Rb_2_GaAgF_6_.

Figure 2a–d illustrates the optimum ground-state energy as a function of unit-cell volume for A_2_GaAgF_6_ (A = Na, K, Rb, Cs), providing good insight into the equilibrium characteristics and structural stability of these fluoride double perovskites. The energy-volume datasets were fitted using the Murnaghan equation of state^34^, facilitating a precise estimate of the equilibrium structural parameters. The optimized unit-cell volumes and lattice constants are presented in Table 1. At equilibrium, the ground-state energies were determined to be − 12,751.95 eV for Na_2_GaAgF_6_, − 11,718.88 eV for K_2_GaAgF_6_, − 11,517.00 eV for Rb_2_GaAgF_6_, and − 11,685.74 eV for Cs_2_GaAgF_6_. The existence of distinct minima in all energy–volume curves affirm the structural stability of the compounds and substantiates the efficacy of the optimization process. Furthermore, the Goldschmidt tolerance factor (τ) was calculated to analyze the geometric stability of these cubic double perovskites, represented in Eq. (4)^35^.

4 $$\tau = \frac{{R}_{A}+ {R}_{F}}{\sqrt{2} \left[\left(\frac{{R}_{Ga }+ {R}_{Ag }}{2}\right)+ {R}_{F}\right]}$$

where R_A_ and R_F_ denote the ionic radii of the A-site cation and the fluorine anion, respectively, R_Ga_ and R_Ag_ align with the ionic radii of Ga and Ag cations. The values of τ that were calculated fell within the acceptable range (0.85—1.1)^36^, hence validating the structural viability and cubic stability of the A_2_GaAgF_6_ compounds, as delineated in Table 1. Additionally, the thermodynamic stability of these DPs was assessed by calculating the formation enthalpies (E_f_), expressed in Eq. (5)^37^.

5 $$E_{f} = \, \left[ {E_{A2GaAgF6} - \, \left( {2E_{A} + \, E_{Ga} + \, E_{Ag} + \, 6E_{F} } \right)} \right]$$

**Table 1:** Optimized structural models and associated thermodynamic stability parameters for A_2_GaAgF_6_ (A = Na, K, Rb, and Cs) comparing with previous study.

Material name	Bandgap, (E_g_)	Lattice constant (Å)	Unit cell volume (Å^3^)	τ	E_f_ (eV/atom)	Ref
Na_2_GaAgF_6_	1.55	8.56	626.49	0.866	- 2.00	This work
K_2_GaAgF_6_	2.203	8.68	654.88	0.946	- 2.03	
Rb_2_GaAgF_6_	2.396	8.79	678.86	0.972	- 2.06	
Cs_2_GaAgF_6_	2.698	8.96	720.19	1.022	- 2.10	
Na_2_AuSbF_6_	1.3	9.15	766.06	0.98	- 2.47	^38^
K_2_AuSbF_6_	1.16	9.21	781.23	1.08	- 2.88	
Li_2_AlAgF_6_	1.86	8.28	567.66	0.82	- 1. 23	^15^
Na_2_InGaF_6_	2.9	8.95	716.92	0.94	- 1.75	^39^

The negative E_f_ values show that the synthesis of these materials is exothermic, implying that energy escapes throughout manufacturing, hence strengthening the intrinsic stability of the materials. The findings together confirm that all studied A_2_GaAgF_6_ (A = Na, K, Rb, and Cs) perovskites display strong cubic symmetry, stable geometric arrangements, and advantageous thermodynamic properties, as outlined in Table 1.

### Phonon examination

**Fig. 3:**
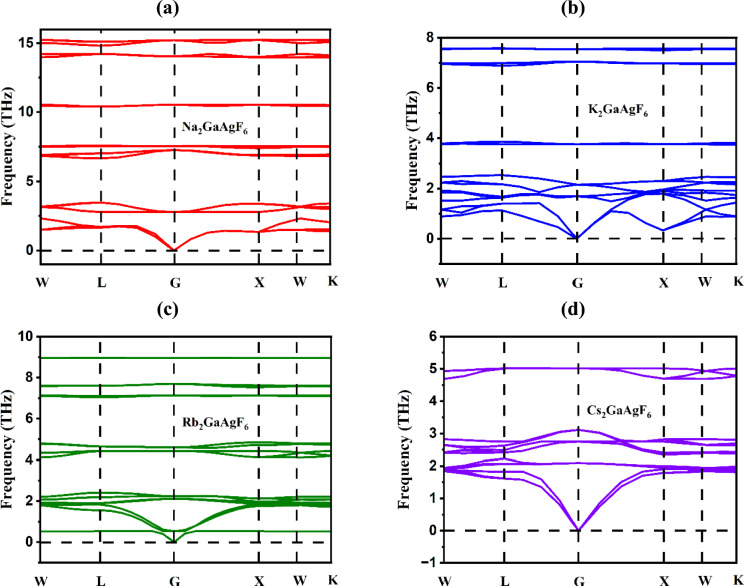
Computed phonon dispersion curves for A_2_GaAgF_6_ (where A = Na, K, Rb, Cs) compounds, demonstrating the dynamic stability of the structures.

The dynamic stability of crystalline materials is essential for their practical use in optoelectronic and solar applications, particularly under realistic working settings where heat fluctuations, mechanical stress, and external disturbances are inevitable. Phonon dispersion analysis serves as an effective microscopic instrument for examining lattice dynamics, interatomic interactions, and vibrational stability. This study estimated the phonon dispersion curves (PDCs) of A_2_GaAgF_6_ (where A = Na, K, Rb, Cs) double perovskites along the high-symmetry directions (W–L–Γ–X–W–K) within the first Brillouin zone, as depicted in Fig. 3. A material is deemed dynamically stable when all phonon modes display positive (real) frequencies across the Brillouin zone, while the existence of imaginary modes signifies lattice instabilities and potential structural phase transitions^40,41^. The calculated phonon spectra for all examined substances exhibit no imaginary phonon modes over the entire Brillouin zone, hence affirming their strong dynamical stability. This signifies that the optimized crystal structures represent genuine minima on the potential energy surface and exhibit resistance to tiny atomic displacements. Comparable phonon-based stability criteria have been extensively utilized in recent investigations of halide and fluoride double perovskites. Nasarullah et al.^42^ proved that dynamically stable double perovskites had phonon spectra devoid of soft modes, validating their structural viability for optoelectronic applications. Similarly, Rehman et al.^29^ underscored that phonon stability serves as a critical determinant for the long-term structural integrity of lead-free perovskite materials, especially in ambient environments. The lack of soft modes in A_2_GaAgF_6_ indicates that these materials are stable at 0 K and are anticipated to preserve structural integrity at limited temperatures, including room temperature, which is crucial for solar device functionality^42^. Materials demonstrating perfect phonon stability at 0 K are typically considered excellent prospects for steady performance under ambient conditions, as thermal vibrations at room temperature are inadequate to destabilize the lattice. The phonon spectrum comprises twelve branches, including three acoustic modes and nine optical modes, in accordance with the four-atom unit cell. The acoustic modes accurately converge to zero frequency at the Γ-point, confirming computational precision, whereas optical modes result from relative atomic oscillations within the lattice. A distinct differentiation between acoustic and optical branches is evident owing to the mass disparity between fluorine anions and A-site cations (Na⁺, K⁺, Rb⁺, Cs⁺). A steady decrease in optical phonon frequencies with increasing cation mass is noted, ascribed to mass-dependent phonon softening, in agreement with other studies on halide perovskites. The phonon dispersion data, in conjunction with the previously proven mechanical stability, affirm that all A_2_GaAgF_6_ compounds exhibit both mechanical and dynamical stability inside the cubic elpasolite structure^43^. The findings align closely with previous theoretical studies on stable double perovskites and indicate that the materials examined are viable candidates for thermally resilient photovoltaic and optoelectronic applications, where long-term stability and dependable room-temperature operation are critical for device performance and longevity.

### Electronic attributes

**Fig. 4:**
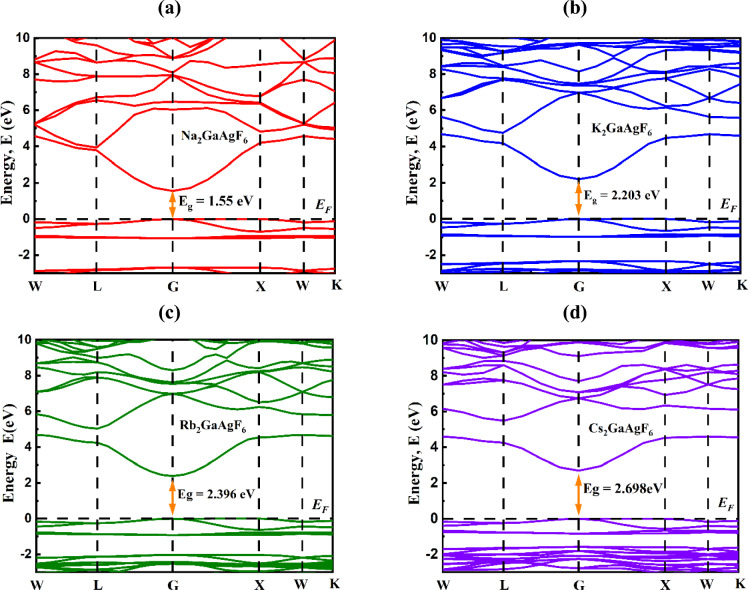
Computed electronic band structures of (**a**) Na_2_GaAgF_6_, (**b**) K_2_GaAgF_6_, (**c**) Rb_2_GaAgF_6_, and (**d**) Cs_2_GaAgF_6_.

The electrical properties of a material are crucial in influencing its efficacy and suitability for PV and optoelectronic applications. Comprehending the band structure and energy gap characteristics yields essential insights into charge carrier dynamics, optical absorption, and electronic transitions inside a material. The calculated electronic band structures of A_2_GaAgF_6_ (where A = Na, K, Rb, and Cs) are depicted in Fig. 4a–d, emphasizing the essential characteristics that dictate their semiconducting properties. First-principles calculations performed inside the GGA-PBE framework demonstrated direct band gaps at the Γ (Gamma) point, confirming that all investigated materials are direct-gap semiconductors. The PBE-GGA exchange–correlation functional is recognized for its persistent underestimation of semiconductor band gap magnitudes; yet it is dependable for accurately reflecting relative electronic trends and qualitatively recreating band structure features. This renders it especially appropriate for comparative analyses and extensive simulations of intricate materials. Although advanced methodologies, such as hybrid functionals (e.g., HSE06) or many-body perturbation techniques (GW approximations), can produce more precise absolute band gap values, their computational demands significantly escalate, particularly when addressing large supercells, defect-laden systems, or doped configurations^44^^,^^45^. Furthermore, these sophisticated techniques may sometimes overstate band gaps or modify critical electronic characteristics, such as carrier effective masses and density of states, resulting in discrepancies with empirically measured outcomes^46^. Consequently, the utilization of the PBE functional in this study represents a deliberate balance between computational efficiency and physical accuracy. This option facilitates the methodical analysis of inherent electrical properties, defect energetics, and band alignment inside the A_2_GaAgF_6_ perovskite structure.

The determined energy band gaps are 1.55 eV for Na_2_GaAgF_6_, 2.203 eV for K_2_GaAgF_6_, 2.396 eV for Rb_2_GaAgF_6_, and 2.698 eV for Cs_2_GaAgF_6_. The indicated band-gap magnitudes imply the capability for absorption of visible to near-infrared light, making these substances potential for solar energy transformation and light-emitting applications^47^. The band structure diagrams illustrate that the Fermi level is indicated by a horizontal dashed line at 0 eV. The valence band maximum (VBM) is situated immediately below this reference, while the conduction band minimum (CBM) is positioned above it, spanning an energy range of approximately −3 eV to + 10 eV. The closeness of the Fermi level to the valence band edge signifies a p-type semiconductor nature, with holes serving as the principal charge carriers. This fact is consistent with prior theoretical and experimental research on lead-free halide double perovskites, hence validating the current computational results (Table 1)^48,49^. A significant consistent rise in the band gap is found with the substitution of the A-site cation from Na to Cs. The band gap increases from 1.55 eV to 2.698 eV, reflecting an almost 74% rise over the series. This evident tendency can be principally ascribed to lattice expansion and diminished ionic polarizability linked to bigger alkali-metal cations. The gradual expansion of the ionic radius of the A-site cation from Na⁺ to K⁺, Rb⁺, and Cs⁺ results in a systematic elongation of the Ga–F and Ag–F bond lengths, as detailed in Table S1 (Supplementary Information). The lattice expansion diminishes the orbital overlap between the Ag-d and F-p states. As a result, the reduced hybridization leads to an expansion of the electronic band gap, consistent with the recognized structure–property correlations in halide perovskite systems^50^.

The primary process results from cation-induced structural distortions, alterations in bond lengths, and variations in lattice polarizability linked to the chemical characteristics of the A-site ions. The results indicate that the electronic band gap of A_2_GaAgF_6_ compounds can be precisely modified via A-site cation substitution, allowing for regulated alteration of their optical absorption threshold and charge-transport properties. This tunability underscores the potential of lead-free perovskites for advanced PV and optoelectronic applications, where environmental sustainability and superior device performance are essential.

**Fig. 5:**
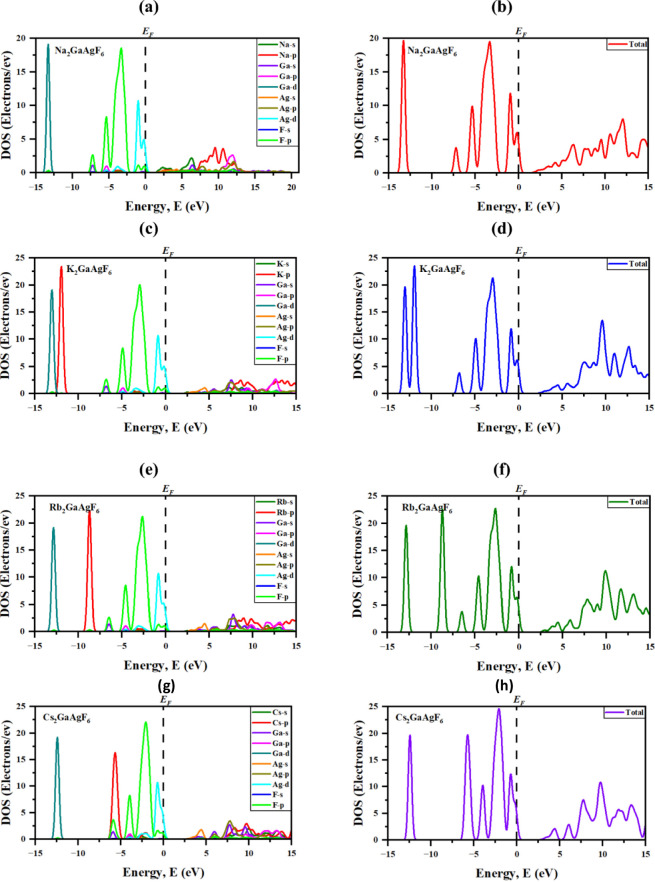
Computed (**a**, **c**, **e**, **g**) partial and (**b**, **d**, **f**, **h**) total DOS for A_2_GaAgF_6_ (A = Na, K, Rb, Cs) compounds, demonstrating the electronic contributions of several atomic orbitals and their significance in shaping the overall electronic structure.

To enhance comprehension of the electronic structure and orbital interactions influencing the behavior of the examined materials, the total and partial density of states (TDOS and PDOS) for A_2_GaAgF_6_ were computed and are illustrated in Fig. 5a–h. In these calculations, the Fermi level was designated as the reference energy at 0 eV. The DOS analysis offers essential insights into the distribution of electronic states and their associated atomic and orbital contributions, which jointly dictate the electrical and optical properties of the compounds. The TDOS spectra illustrates the cumulative contribution of all atomic orbitals to the energy bands, emphasizing the relative density of states in both the valence band (VB) and conduction band (CB) regions. Concurrently, the PDOS plots provide a more nuanced view by delineating the precise contributions of various atomic orbitals to the band structure. This study facilitates a clear comprehension of bonding features, hybridization effects, and electronic transitions among various energy levels. The PDOS profiles indicate that the VB is predominantly formed by contributions from the F-p and Ag-d orbitals, with minimal involvement from the Ga-p states. The robust hybridization between Ag-d and F-p orbitals signifies the establishment of covalent bonding contacts inside the perovskite lattice, which is crucial in determining the VB dispersion and hole mobility^51^. The Ga-p orbitals, while less prominent, contribute to the expansion of the upper valence area, indicating a weak p-d connection that improves overall band mixing. In contrast, the CB predominantly originates from the Ag–s orbitals, with minor contributions from the A-p and Ga-s orbitals. The DOS study indicates that the F-p and Ag-d orbitals predominate in the valence region, whereas the Ag–s orbitals control the conduction region, demonstrating a distinct split between bonding and anti-bonding states. These findings offer essential insight into the structure–property relationship of A_2_GaAgF_6_ perovskites and highlight their potential as adjustable lead-free semiconductors for PV and optoelectronic applications.

### Mechanical properties

An equilibrium between mechanical stability and elastic anisotropy guarantees that the material preserves structural integrity while effectively adjusting to diverse operational conditions^52^. Mechanically strong materials enhance long-term durability and reliability, whereas anisotropy facilitates direction-dependent adjustment of physical properties, improving performance in various functional contexts. Consequently, the mechanical characteristics of A_2_GaAgF_6_ double perovskites were methodically assessed by calculating their elastic constants (ECs). In cubic systems, three distinct elastic constants C_11_, C_12_, and C_44_ characterize the essential mechanical response of the crystal lattice. The constants enumerated in Table 2 were utilized to assess the mechanical stability of the examined compounds in line with the Born-Huang stability criterion for cubic crystals^53^, articulated in Eq. (6).

6 $$\left( {C_{11} - \, C_{12} } \right) \,> \, 0; \, C_{11}> \, 0; \, C_{44}> \, 0; \, \left( {C_{11} + \, 2C_{12} } \right) \,> \, 0$$

**Table 2:** The computed elastic constants for cubic A_2_GaAgF_6_ perovskites.

SL NO	Material name	C_11_	C_12_	C_44_	C_11_-C_12_	C_11_ + 2C_12_	C_12_-C_44_
1	Na_2_GaAgF_6_	79.177	22.467	19.89	56.71	124.111	2.577
2	K_2_GaAgF_6_	76.246	25.655	22.534	50.591	127.556	3.121
3	Rb_2_GaAgF_6_	71.705	28.333	23.362	43.372	128.371	4.971
4	Cs_2_GaAgF_6_	68.923	31.6	26.146	37.3	132.123	5.454

All examined A_2_GaAgF_6_ compounds fulfill these criteria, hence affirming their mechanical stability under equilibrium conditions. C_11_ denotes the resistance to longitudinal strain (axial deformation), whilst C_12_ and C_44_ signify the resistance to transverse and shear deformations, respectively. The primary elastic constants were subsequently employed to calculate secondary mechanical moduli, specifically the bulk modulus (B), shear modulus (G), Young’s modulus (Y), and Poisson’s ratio (v), via the following relationships in Eqs. (7–10)^54,55^.

7 $$B= \frac{{C}_{11}+2 {C}_{12}}{3}$$

8 $$G= \frac{1}{2 } \left[ \frac{{C}_{11}- {C}_{12}+3{C}_{44}}{5}+ \frac{5 {C}_{44} \left({C}_{11}- {C}_{12}\right)}{4{C}_{44}+3\left({C}_{11}- {C}_{12}\right)}\right]$$

9 $$Y= \frac{9BG}{(3B+G)}$$

10 $$v= \frac{3B-2G}{2(3B+G)}$$

The mechanical characteristics shown in Table 3 indicate a distinct trend: an increase in the molecular mass of the A-site cation correlates with a decrease in the overall elastic constants and associated moduli. This observation indicates that the replacement of lighter alkali cations with heavier one’s results in a softer and more compressible lattice structure. Specifically, Na_2_GaAgF_6_ demonstrates the highest bulk modulus, signifying superior incompressibility and rigidity relative to K_2_GaAgF_6_, Rb_2_GaAgF_6_, and Cs_2_GaAgF_6_. Significantly, the values of B surpass the corresponding values of G for all compounds, indicating that these materials exhibit greater resistance to volumetric compression than to shape deformation. Likewise, the elevated values of Y for Na_2_GaAgF_6_ indicate its enhanced stiffness and more robust interatomic bonding in comparison to its heavier counterparts. This mechanical rigidity is beneficial for preserving lattice stability under mechanical stress, a sought-after characteristic for energy conversion and electrical applications^56^. The Poisson’s ratio (ν), indicating a material’s capacity for transverse deformation under axial load, offers additional understanding of bonding characteristics and ductility. Generally, ν < 0.26 signifies brittle behavior, while ν > 0.26 indicates ductile behavior^57^. All examined perovskites demonstrate ν values over 0.26, affirming their ductile characteristics and indicating their capacity to endure mechanical deformation without fracture, an indispensable attribute for device manufacturing and mechanical flexibility. Pugh’s ratio (B/G) is a further measure of ductility, with B/G > 1.75 indicating ductile behavior and B/G < 1.75 indicating brittle behavior^58^. The computed B/G ratios for all A_2_GaAgF_6_ combinations surpass this threshold, hence reinforcing their ductile and flexible nature (Table 3). The Zener anisotropy factor (A) was calculated to evaluate the extent of elastic anisotropy as following Eq. (11)^59^.

**Table 3:** First-principles simulations of the mechanical characteristics of cubic A_2_GaAgF_6_ (A = Na, K, Rb, Cs) perovskites indicate trends in stiffness and ductility based on different alkali elements.

SL NO	Material name	B	G	E	v	B/G	A
1	Na_2_GaAgF_6_	51.37	18.68	47.513	0.39	2.751	2.829
2	K_2_GaAgF_6_	47.186	16.195	44.081	0.354	2.913	0.277
3	Rb_2_GaAgF_6_	43.457	14.668	39.555	0.338	2.962	0.160
4	Cs_2_GaAgF_6_	39.033	10.989	30.139	0.321	3.552	0.021

11 $$A= \frac{2{C}_{44}}{{C}_{11} - {C}_{12}}$$

A fully isotropic crystal has A = 1, whereas variations signify anisotropic behavior. The acquired A values deviate from unity for all A_2_GaAgF_6_ materials, indicating significant elastic anisotropy. This anisotropy is beneficial for practical applications, since it facilitates direction-dependent mechanical flexibility and stress accommodation. The anisotropic behavior demonstrates that Na_2_GaAgF_6_ displays the highest level of anisotropy within the series, succeeded by compounds based on K, Rb, and Cs. This anisotropic flexibility renders these materials interesting options for sophisticated engineering and energy-harvesting systems that require both mechanical durability and structural adaptation.

### Optical properties

The optical characteristics of materials provide crucial understanding of their prospective uses in PV, optoelectronic, and photonic fields, as they dictate the fundamental processes of photon-electron interactions, electronic excitation, energy dissipation, and light propagation within the crystal lattice. To thoroughly evaluate these behaviors, many critical optical properties of the A_2_GaAgF_6_ (A = Na, K, Rb, Cs) double perovskites were analyzed within the photon energy spectrum of 0—40 eV.

**Fig. 6:**
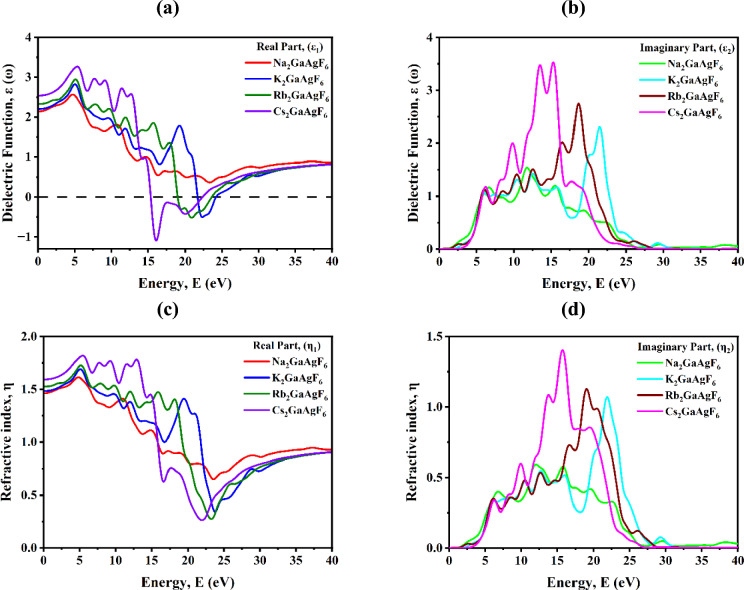
Calculated optical parameters for A_2_GaAgF_6_: (**a**) ε_1_, (**b**) ε_2_, (**c**) n, and (**d**) k.

The dielectric function ε(ω), characterized by its real and imaginary components, delineates a material’s polarization in reaction to an applied optical field. The ε_1_(ω) delineates the dispersion and retention of electromagnetic energy, while the ε_2_(ω) pertains to absorption resulting from interband electronic transitions^60^. Figure 6a depicts the variation of ε_1_(ω) for A_2_GaAgF_6_ compounds. The static dielectric constants ε_1_(0) were measured as 2.14, 2.21, 2.33, and 2.53 for Na_2_GaAgF_6_, K_2_GaAgF_6_, Rb_2_GaAgF_6_, and Cs_2_GaAgF_6_, respectively. The gradual rise in ε_1_(0) with heavier A-site cations indicates a consistent improvement in electronic polarizability. This is due to the larger ionic radii of K⁺, Rb⁺, and Cs⁺ in comparison to Na⁺, resulting in increased lattice relaxation and electron cloud deformability under an optical field. The bigger cations cause minor lattice distortions that enhance dipole moments, thereby elevating the real portion of the dielectric function, which aligns with the known dielectric properties of polar materials^61^^,^^39^. As photon energy increases, ε_1_(ω) initially ascends, attains a peak, and then diminishes at elevated energies. This pattern signifies that the materials have normal dispersion at low frequencies and anomalous dispersion in resonance areas, where optical transitions are prominent. The peak values of ε_1_(ω) are recorded at 2.56, 2.82, 2.94, and 3.26 for Na_2_GaAgF_6_, K_2_GaAgF_6_, Rb_2_GaAgF_6_, and Cs_2_GaAgF_6_, respectively, indicating that Cs_2_GaAgF_6_ exhibits the most robust polarization response among the examined compounds. Figure 6b illustrated the ε₂(ω), indicates the extent of optical energy absorption by the material via interband transitions from filled valence states to vacant conduction states. The initial significant absorption peaks in ε₂(ω) are observed at 11.8, 21.53, 18.6, and 15.36 eV for Na_2_GaAgF_6_, K_2_GaAgF_6_, Rb_2_GaAgF_6_, and Cs_2_GaAgF_6_, respectively, with corresponding intensities of 1.54, 2.29, 2.74, and 3.51. The peak positions denote the energy thresholds at which photon absorption is maximized, relating to transitions predominantly between Ag-d and F-p states at the VBM, and A-s and Ga-s states at the CBM. The pronounced ε₂(ω) responses signify substantial electronic polarization, underscoring their applicability in visible to ultraviolet optical domains.

The refractive index (n(ω)) and extinction coefficient (k(ω)) offer additional insights into the propagation and attenuation of light within the material. Figure 6c depicts that the static refractive indices at zero frequency are 1.462, 1.483, 1.527, and 1.592 for Na_2_GaAgF_6_, K_2_GaAgF_6_, Rb_2_GaAgF_6_, and Cs_2_GaAgF_6_, respectively. The values exhibit a monotonically increasing trend with cation size, mirroring the pattern observed in ε_1_(0), as anticipated from the fundamental relationship n^2^(0) = ε_1_(0)^62^. The rising trend in refractive index indicates that the optical density of the materials increases with greater A-site cations, leading to diminished photon propagation and enhanced light confinement inside the medium. Moreover, as the n(ω) is inversely related to the band gap, the observed sequence (Na_2_GaAgF_6_ to Cs_2_GaAgF_6_) illustrates the previously mentioned band gap reduction^63^. The peak values of n(ω) are recorded at 1.61, 1.68, 1.72, and 1.81 for Na_2_GaAgF_6_, K_2_GaAgF_6_, Rb_2_GaAgF_6_, and Cs_2_GaAgF_6_, corresponding to photon energies of 4.8, 5.32, 5.21, and 5.51 eV, respectively. The k(ω), seen in Fig. 6d, denotes the exponential attenuation of light intensity as it passes through the material due to absorption. The initial k(ω) peaks are observed at 2.06, 2.8, 3.05, and 3.8 eV for the various chemicals, signifying the commencement of robust absorption and denoting their change from transparent to absorptive states. The intensity and location of these peaks indicate that optical activity predominantly arises around the band edge region, aligning with semiconducting characteristics^64^.

**Fig. 7:**
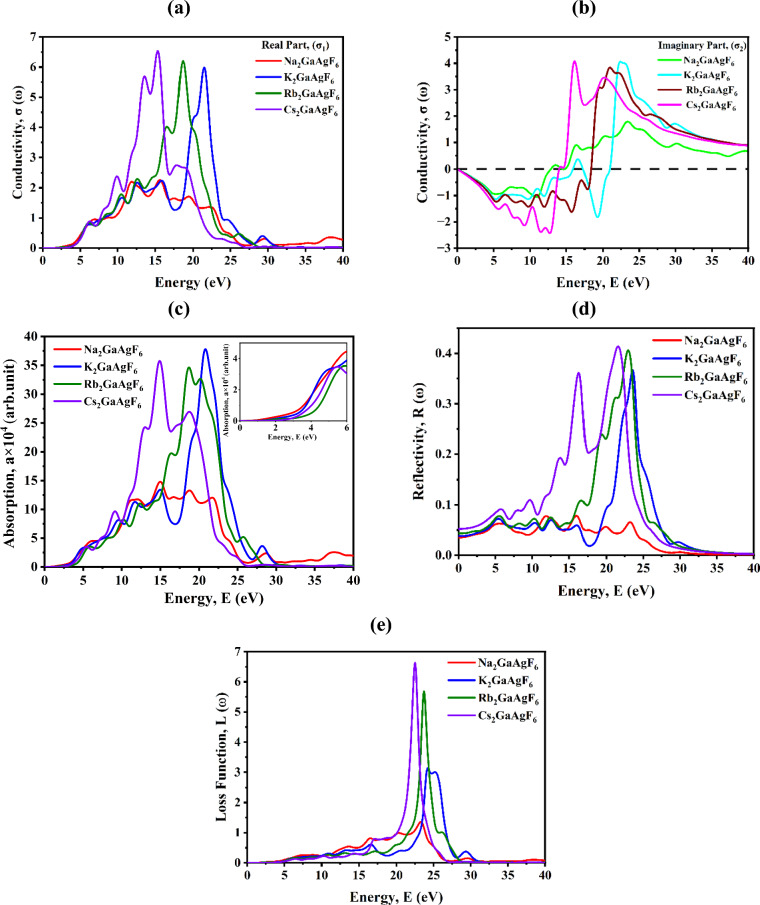
Evaluated optical functions of A_2_GaAgF_6_, such as: (**a**, **b**) photoconductivity (real and imaginary parts), (**c**) absorption coefficient, (**d**) reflectivity, and (**e**) loss function.

The optical conductivity (σ(ω)), depicted in Fig. 7a,b, indicates the rate at which electrons acquire energy from the optical field and traverse the crystal lattice. In all A_2_GaAgF_6_ compounds, σ(ω) demonstrates a pronounced increase just above the band gap, succeeded by several resonant peaks ranging from 3 to 20 eV. The peaks result from consecutive interband electronic transitions, aligning with the previously mentioned DOS characteristics (Fig. 5). The elevated σ(ω) values indicate effective photoinduced charge transport, essential for optimal performance in photodetectors and solar absorbers^65^.

The absorption coefficient (α(ω)) measures the efficacy of a material in absorbing incident photons of different energies, which is essential for PV efficiency. Figure 7c illustrates that all chemicals exhibit significant absorption throughout a broad spectral range of 0–40 eV, with notable peaks in the visible spectrum. The determined maximum α(ω) values are 1.474 × 10^5^ cm^−1^, 3.78 × 10^5^ cm^−1^, 3.46 × 10^5^ cm^−1^, and 3.574 × 10^5^ cm^−1^ for Na_2_GaAgF_6_, K_2_GaAgF_6_, Rb_2_GaAgF_6_, and Cs_2_GaAgF_6_, respectively. The greater value of α(ω) than 10^5^ cm^−1^ in the visible spectrum indicates exceptional light-harvesting capability, like or exceeding that of traditional lead-halide perovskites^66^. The initiation of absorption aligns closely with the estimated band gap of each molecule, validating that the photon energy necessary for electronic excitation directly corresponds to the basic transition energies. Additionally, a significant red shift in the absorption edge occurs from around ~ 2.68 eV (Cs_2_GaAgF_6_) to around ~ 1.54 eV (Na_2_GaAgF_6_), signifying a gradual redshift in the optical absorption edge as the A-site ionic radius diminishes. The inset of Fig. 7c presents a magnified image of the low-energy spectral range (0–6 eV), corroborating the transition toward the visible spectrum. The redshift arises from increased orbital overlap and diminished lattice distortion in lighter alkali compositions, resulting in a narrowed band gap and greater visible-light absorption. This property is particularly advantageous for solar energy conversion, as it enables the effective utilization of lower-energy photons.

The reflectivity spectra (R(ω)), illustrated in Fig. 7d, demonstrates comparatively low reflection at diminished photon energies 0.0353, 0.0379, 0.0435, and 0.0523 for Na_2_GaAgF_6_, K_2_GaAgF_6_, Rb_2_GaAgF_6_, and Cs_2_GaAgF_6_, respectively. As the energy of photons increases, reflectivity gradually increases, attaining peak values of 0.0767, 0.3624, 0.4023, and 0.4133, respectively. The little initial reflectivity is advantageous for PV applications, facilitating the penetration of incident light into the material instead of reflecting it away. The measured reflectivity maxima align with areas where ε_1_(ω) is negative, indicating plasmonic resonances typical of metallic optical behavior at elevated photon energy^67^.

The energy loss function (L(ω)), seen in Fig. 7e, elucidates the energy dissipation of rapid electrons as they navigate the material and engage with its electronic system. L(ω) characterizes the collective oscillation of conduction electrons (plasmons) and quantifies the energy loss per unit path length resulting from these excitations. The spectra of L(ω) for all four compounds display low values (below 0.40) up to 3.5 eV, indicating minor plasmonic losses and insignificant electron scattering beneath the optical band gap. The lack of significant peaks in the low-energy range indicates superior optical transparency and negligible internal energy dissipation. The concurrent reduction in R(ω) and L(ω) at elevated photon energies signifies that light absorption prevails over reflection, thereby facilitating effective photon-to-electron conversion.

The aggregate optical characteristics featuring high absorption, minimal reflection, robust dielectric response, and insignificant energy loss validate that A_2_GaAgF_6_ (A = Na, K, Rb, Cs) compounds exhibit exceptional optical and electronic synergy. These characteristics render viable lead-free substitutes for conventional perovskites in PV, optoelectronic, and photonic device frameworks.

### Device structure and material parameters

**Fig. 8:**
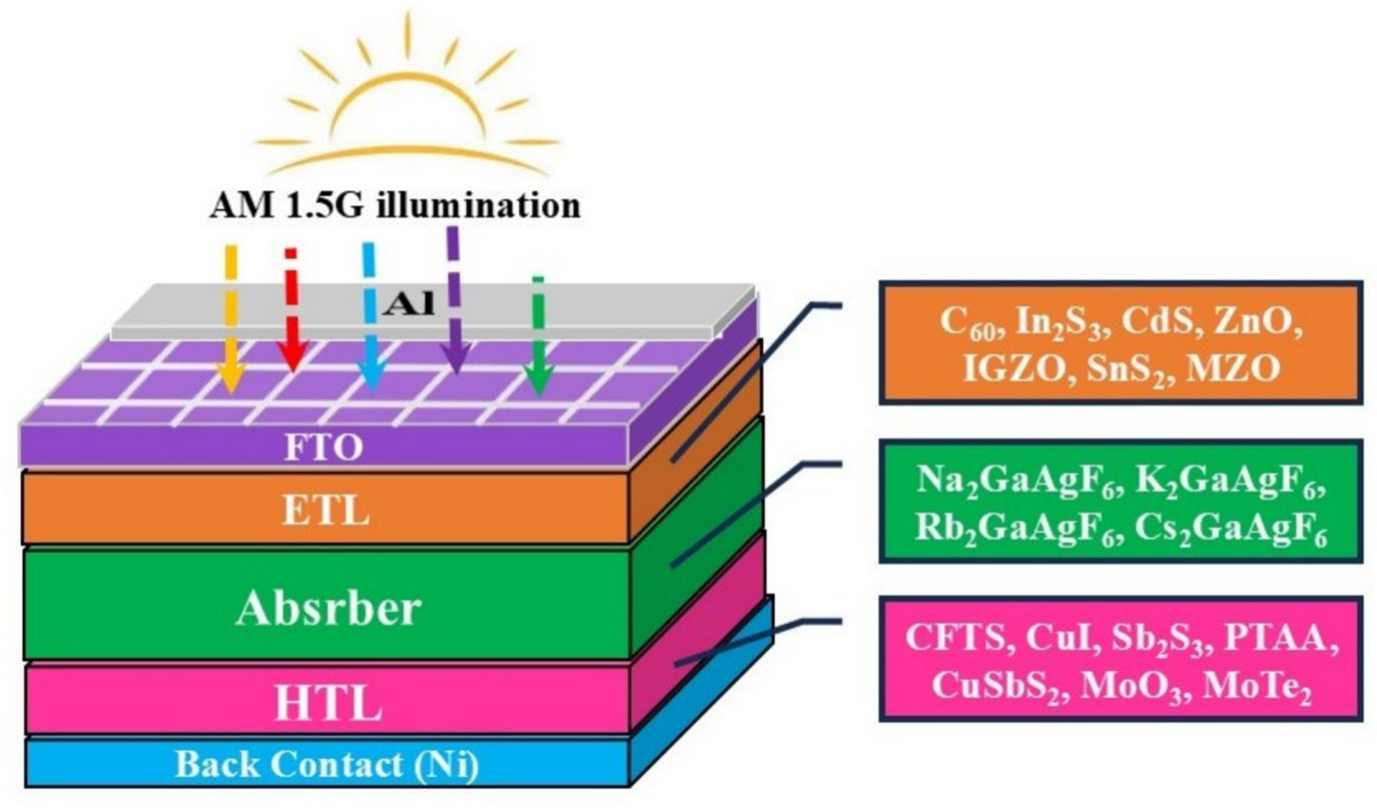
Device architecture: front contact/FTO/ETL/A_2_GaAgF_6_/HTL/back contact with n-i-p configuration.

The conceptual design of the lead-free DPSC structure is shown in Fig. 8. This structure is comprised of an absorber layer of A_2_GaAgF_6_ (Na, K, Rb, and Cs) that is integrated with ETL, HTL, and a back contact. The solar cell employs a n–i–p arrangement, wherein the absorber layer is composed of A_2_GaAgF_6_ and is characterized as intrinsic due to the absence of intentional doping. The absorber demonstrates a quasi-intrinsic nature with a minimal p-type background resulting from native defect states, a phenomenon frequently noted in wide-bandgap double perovskites. In contrast to traditional p–n junction devices, the n–i–p architecture provides superior sensitivity to long-wavelength photons owing to its extensive intrinsic (or near-intrinsic) area^68,69^. This arrangement includes an inherent layer that generates a depletion area extending deeply into the device, enhancing the penetration of long-wavelength photons. This results in increased electron–hole pair formation both within and beyond the depletion zone, leading to improved QE due to the effective generation and separation of charge carriers across the expanded depletion region. The double heterostructure of A_2_GaAgF_6_ is crucial for capturing charge carriers and photons, hence markedly improving its photon absorption capability. Moreover, it establishes ohmic connections with the heavily doped HTL and ETL interfaces. The HTLs utilized in the device assembly comprise CFTS, CuI, Sb_2_S_3_, PTAA, CuSbS_2_, MoO_3_, and MoTe_2_, with the back and the front contact consisting of nickel (Ni) and aluminum (Al), respectively. The ETL materials considered include C_60_, In_2_S_3_, CdS, ZnO, IGZO, SnS_2_, and MZO, with alkali-based halide perovskites (A_2_GaAgF_6_) utilized as the absorber layer. A comprehensive arrangement of 196 device combinations was simulated by altering seven ETL and seven HTL materials, structured in the architecture: Al/FTO/ETL/A_2_GaAgF_6_/HTL/Ni, to assess interfacial compatibility and performance trends.

The simulation input parameters for the absorber layers and ETLs are summarized in Table 4, whereas the corresponding parameters for FTO and HTLs are provided in Table 5. The interfacial layer parameters are listed in Table 6. The efficacy of different DPSC designs was assessed with the SCAPS-1D simulation software under typical operating conditions, which included the AM1.5G sun spectrum, a frequency of 1 MHz, and an ambient temperature of 300 K. A fixed series resistance (R_S_) of 0.5 Ω·cm^2^ and a shunt resistance (R_Sh_) of 10^5^ Ω·cm^2^ were utilized, in accordance with values documented in prior research^70,71^.

**Table 4:** Input parameters of ETLs and absorber layers^72–74^.

Parameter	Na_2_GaAgF_6_	K_2_GaAgF_6_	Rb_2_GaAgF_6_	Cs_2_GaAgF_6_	C_60_	In_2_S_3_	CdS	ZnO	IGZO	SnS_2_	MZO
T (nm)	1000	1000	1000	1000	50	50	50	50	50	50	50
E_g_ (eV)	1.55	2.203	2.396	2.698	1.7	2.1	2.4	3.3	3.05	2.24	3.5
χ (eV)	4.3	4.05	3.64	3.26	3.9	4	4.2	4	4.16	4.24	4.05
εr (eV)	2.12	2.2	2.33	2.54	4.2	13.5	10	9	10	10	8.5
N_C_ (cm^−3^)	4.74 × 10^18^	7.91 × 10^18^	6.32 × 10^18^	6.64 × 10^18^	8.0 × 10^19^	1.8 × 10^19^	2.2 × 10^18^	3.7 × 10^18^	5 × 10^18^	2.2 × 10^18^	2.2 × 10^18^
N_V_ (cm^−3^)	1.2 × 10^19^	1.42 × 10^19^	1.39 × 10^19^	1.55 × 10^19^	8.0 × 10^19^	4 × 10^13^	1.8 × 10^19^	1.8 × 10^19^	5 × 10^18^	1.8 × 10^19^	1.8 × 10^19^
u_n_ (cm^2^V^−1^ s^−1^)	15.5	5.25	2.65	1.55	0.08	400	100	100	15	50	100
u_p_ (cm^2^V^−1^ s^−1^)	7.75	2.6	1.55	1.025	0.0035	210	25	25	0.1	50	25
N_A_ (cm^−3^)	1 × 10^17^	1 × 10^17^	1 × 10^17^	1 × 10^17^	0	0	0	0	0	0	0
N_D_ (cm^−3^)	0	0	0	0	1 × 10^18^	1 × 10^18^	1 × 10^18^	1 × 10^18^	1 × 10^18^	1 × 10^18^	1 × 10^18^
N_t_ (cm^−3^)	1 × 10^14^	1 × 10^14^	1 × 10^14^	1 × 10^14^	1 × 10^15^	1 × 10^15^	1 × 10^15^	1 × 10^15^	1 × 10^15^	1 × 10^15^	1 × 10^15^

**Table 5:** Input parameters of FTO and different HTLs^75–77^.

Parameters	FTO	CFTS	CuI	Sb_2_S_3_	PTAA	CuSbS_2_	MoO_3_	MoTe_2_
T (nm)	100	100	100	100	100	100	100	100
E_g_ (eV)	3.6	1.87	3.1	1.7	3.3	1.58	3	1.29
χ (eV)	4	3.3	2.1	3.7	2.3	4.2	2.3	4.2
ɛ_r_ (eV)	9	9	6.5	7.08	9	14.6	18	13
Nc (cm^−3^)	2.2 × 10^18^	2.2 × 10^18^	2.8 × 10^19^	2 × 10^19^	1 × 10^21^	2 × 10^18^	1 × 10^19^	4 × 10^16^
Nv (cm^−3^)	1.8 × 10^19^	1.8 × 10^19^	1 × 10^19^	1 × 10^19^	1 × 10^21^	1 × 10^19^	2.2 × 10^18^	3 × 10^18^
μ_n_ (cm^2^V^−1^S^−1^)	100	21.98	100	9.8	1	49	210	110
μ_h_ (cm^2^V^−1^S^−1^)	25	21.98	43.9	10	40	49	210	426
N_A_ (cm^−3^)	0	1 × 10^20^	1 × 10^20^	1 × 10^18^	1 × 10^20^	1 × 10^18^	1 × 10^20^	1 × 10^19^
N_D_ (cm^−3^)	1 × 10^19^	0	0	0	0	0	0	0
N_t_ (cm^−3^)	1 × 10^14^	1 × 10^15^	1 × 10^15^	1 × 10^15^	1 × 10^15^	1 × 10^15^	1 × 10^15^	1 × 10^15^

**Table 6:** Interface parameters used in these DPSCs.

Interface	σ_e_ (cm^2^)	σ_h_ (cm^2^)	E_r_	Defect types	Energetic distribution	N_t_ (cm^−2^)
HTL/A_2_GaAgF_6_	10^–19^	10^–19^	0.6	neutral	single	10^12^
A_2_GaAgF_6_/ETL						

### Optimization of ETL and HTL

**Fig. 9:**
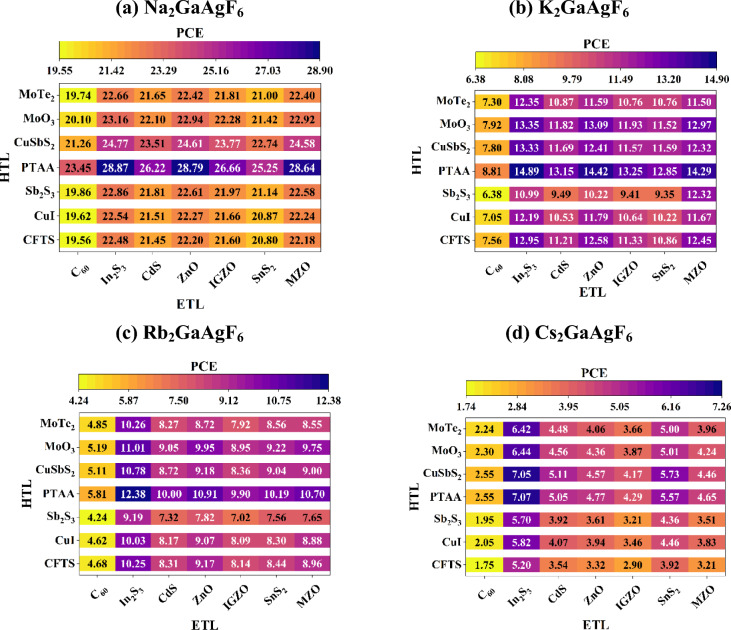
Changes in PCE for (**a**) Na_2_GaAgF_6_, (**b**) K_2_GaAgF_6_, (**c**) Rb_2_GaAgF_6_, and (**a**) Cs_2_GaAgF_6_ solar cells with different HTLs and ETLs.

The photovoltaic efficacy of A_2_GaAgF_6_-based solar cells is significantly influenced by the synergistic interaction of ETLs and HTLs, which serve as charge-selective interfaces and crucially govern carrier extraction and recombination. This study extensively analyzed seven ETLs and HTLs to clarify their impact on device functioning. ETL establishes a thermodynamically advantageous route for photogenerated electrons from the absorber to the FTO front contact, while concurrently serving as a barrier to hole transport. Optimal conduction band alignment between the ETL and absorber facilitates swift electron extraction, minimizing electron buildup at the absorber/FTO interface and mitigating interfacial and bulk recombination losses^78^. Furthermore, ETLs characterized by elevated electron mobility and reduced defect densities diminish carrier entrapment and augment quasi-Fermi level splitting, thereby immediately enhancing V_OC_ and FF. The HTL selectively collects holes from the absorber’s VB and facilitates their transit to the back electrode, while energetically obstructing electrons. Effective hole extraction diminishes charge accumulation within the absorber, thereby decreasing the likelihood of bimolecular and trap-assisted recombination^79^. Moreover, both ETLs and HTLs facilitate interfacial passivation by diminishing surface defect states, which is crucial for minimizing non-radiative recombination in absorbers. This investigation evaluates the efficacy of SCs employing several ETLs including C_60_, In_2_S_3_, CdS, ZnO, IGZO, SnS_2_, and MZO, paired with seven different HTLs: CFTS, CuI, Sb_2_S_3_, PTAA, CuSbS_2_, MoO_3_, and MoTe_2_. Figure 9a–d illustrates the impact of each ETL on PCE for various HTLs in combination with A_2_GaAgF_6_-based SCs. Figure 9a depicts the PCE across several ETLs and HTLs for a device utilizing Na_2_GaAgF_6_. Among the ETLs, In_2_S_3_ had the best PCE of 28.87%, while C_60_ achieved the lowest PCE at 23.45% when paired with PTAA as HTL. Other ETLs, such as CdS, ZnO, IGZO, SnS_2_, and MZO, exhibited PCE values between 25.25% and 28.79% across different configurations. In configurations utilizing the K_2_GaAgF_6_ absorber, the PCE values ranged from 9.35% to 14.89%, except for C_60_, which exhibited the lowest PCE, varying from 6.38% to 8.81% across all ETL and HTL combinations, as portrayed in Fig. 9b. In the assessment of Rb_2_GaAgF_6_ absorber, C_60_ exhibited the lowest PCE at 4.24%, whereas In_2_S_3_ attained the greatest PCE of 12.38%, as presented in Fig. 9c. Among all the ETLs, In_2_S_3_ consistently exhibited superior overall performance. Additionally, Fig. 9d illustrates a performance comparison of the Al/FTO/ETL/Cs_2_GaAgF_6_/HTL/Ni device architecture, wherein ETLs such as In_2_S_3_, CdS, ZnO, IGZO, SnS_2_, and MZO sustained PCEs of 2.90%, whilst the C_60_-based ETL exhibited a markedly inferior efficiency, below 2.55%.

This study thoroughly evaluated the performance of seven different HTLs in conjunction with seven ETLs, emphasizing their efficacy. When combined with the PTAA HTL, the ETLs exhibited robust performance, with PCE values spanning from 23.45% to 28.87% for Na_2_GaAgF_6_, 8.81% to 14.89% for K_2_GaAgF_6_, 5.81% to 12.38% for Rb_2_GaAgF_6_, and 2.55% to 7.07% for Cs_2_GaAgF_6_. The HTLs such as CFTS, CuI, Sb_2_S_3_, CuSbS_2_, MoO_3_, and MoTe_2_ were identified as forming effective combinations with the A_2_GaAgF_6_ absorber. The integration of CFTS and Sb_2_S_3_ HTLs with each ETL showed diminished efficiency. Furthermore, a thorough band alignment analysis was performed to quantitatively assess the appropriateness of several transport layer materials, using conduction band offset (CBO) and valence band offset (VBO) factors with Na_2_GaAgF_6_ absorber. The findings of this investigation are represented in Tables 7 and 8. The ideal CBO for ETL materials should be between 0 to 0.3 eV to facilitate electron extraction and restrict hole leakage into the ETL^80,81^. A positive VBO guarantees efficient hole blocking at the ETL/absorber interface, whereas a near-zero or marginally negative VBO promotes hole extraction in HTLs^82^. The band alignment analysis indicates that In_2_S_3_ offers a suitable CBO value of 0.1—0.2 eV for electron transport, whereas PTAA presents the most excellent VBO of −0.05 eV for hole extraction among the tested HTL materials. The calculations of band offsets provide essential theoretical insights for choosing transport layer materials that reduce interfacial recombination and enhance charge extraction efficiency. In this context, PTAA and In_2_S_3_ are selected as HTL and ETL for further analysis due to their advantageous band alignment, elevated absorption coefficient and excellent optoelectronic properties.

**Table 7:** Analysis of band alignment for ETL/Na_2_GaAgF_6_.

Materials	E_f_ (eV)	E_g_ (eV)	CBO (eV)	VBO (eV)
C_60_	3.9	1.7	0.2	−0.05
In_2_S_3_	4	2.1	0.1	0.45
CdS	4.2	2.4	−0.1	0.95
ZnO	4	3.3	0.1	1.65
IGZO	4.16	3.05	−0.06	1.56
SnS_2_	4.24	2.24	−0.14	0.83
MZO	4.05	3.5	0.05	1.9

**Table 8:** Analysis of band alignment for HTL/Na_2_GaAgF_6_.

Materials	E_f_ (eV)	E_g_ (eV)	CBO (eV)	VBO (eV)
CFTS	3.3	1.87	0.8	−0.48
CuI	2.1	3.1	2	−0.45
Sb_2_S_3_	3.7	1.7	0.4	−0.25
PTAA	2.3	3.3	1.8	−0.05
CuSbS_2_	4.2	1.58	−0.1	0.13
MoO_3_	2.3	3	1.8	−0.35
MoTe_2_	4.2	1.29	−0.1	−0.16

### Impact of front and rear contacts on cell efficacy

**Fig. 10:**
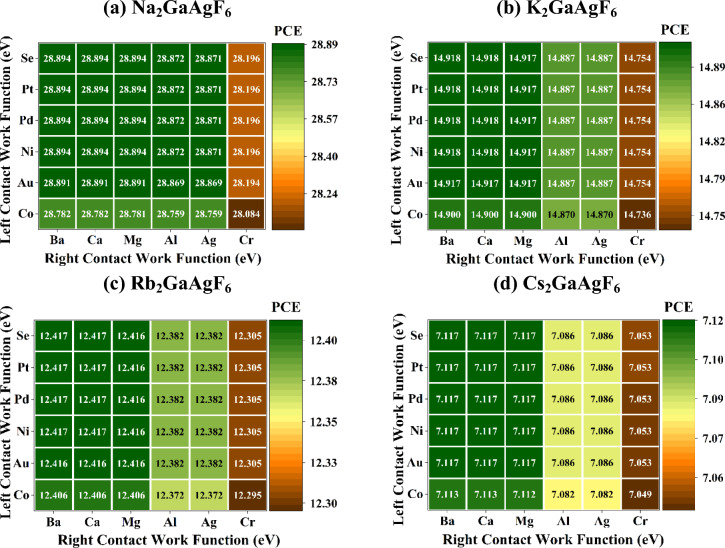
Effect of contact on the device PCE with (**a**) Na_2_GaAgF_6_, (**b**) K_2_GaAgF_6_, (**c**) Rb_2_GaAgF_6_, and (**a**) Cs_2_GaAgF_6_ absorber based DPSCs.

Electrical contacts are crucial in determining the performance of A_2_GaAgF_6_ (A = Na, K, Rb, Cs)-based solar cells, as they regulate effective charge extraction, carrier transport, and recombination suppression. The selection of proper contact materials is crucial to guarantee minimal resistive losses, suitable band alignment with the charge transport layers, and enduring structural and chemical compatibility with the absorber^83^. Figure 10a–d demonstrates the impact of various front and rear metal contacts on power conversion efficiency (PCE), with six front and rear contact materials rigorously analyzed, yielding 36 distinct contact combinations. The combination of nickel (Ni) as the rear (left) contact and aluminum (Al) as the front (right) contact exhibited superior performance across all device topologies. The elevated work function of Ni (~ 5.15 eV) facilitates optimal energy alignment with HTL, namely PTAA, hence promoting efficient hole extraction and mitigating back-electron recombination at the rear interface. Furthermore, Ni establishes a robust ohmic contact with the HTL and demonstrates exceptional thermal and chemical stability, mitigating interfacial deterioration and improving device reliability^84^. Conversely, aluminum, possessing a moderate work function (~ 4.2 eV), aligns efficiently with ETL notably In_2_S_3_ facilitating swift electron collection and transportation to the external circuit. In this design, Al functions as a front metal grid contact deposited on the FTO conductive layer, acting as both an electrical conductor and optical reflector. This grid reduces series resistance and promotes consistent current distribution while enabling light to pass through the FTO layer to the absorber.

The superior conductivity, lightweight characteristics, cost-efficiency, and reflective attributes of aluminum further augment device efficiency by enhancing photon recycling inside the absorber layer. The Ni/Al contact combination guarantees equitable charge extraction, reduced energy barriers, and diminished interfacial recombination. Thus, the Ni–Al configuration produced the best PCEs, about 28.87% for Na_2_GaAgF_6_, 14.89% for K_2_GaAgF_6_, 12.38% for Rb_2_GaAgF_6_, and 7.09% for Cs_2_GaAgF_6_ illustrating its efficacy as an optimum electrode pairing for high-performance, lead-free, and stable double perovskite solar cells.

### Band diagram

**Fig. 11:**
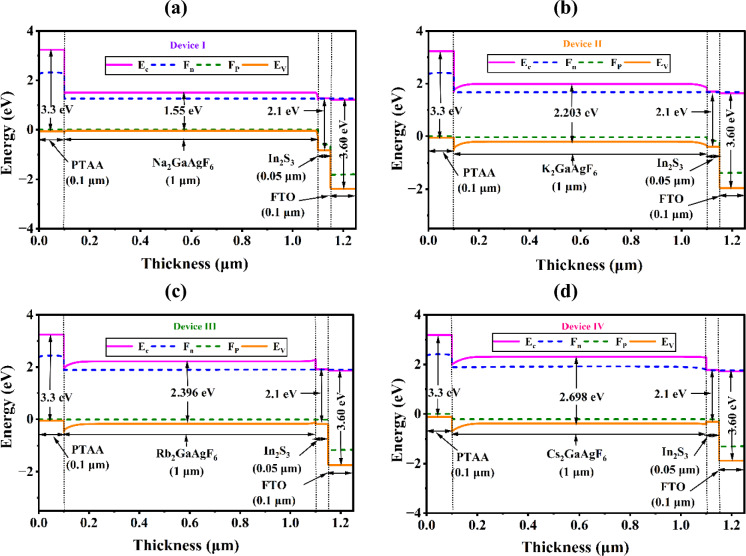
Band Diagram with (**a**) Na_2_GaAgF_6_, (**b**) K_2_GaAgF_6_, (**c**) Rb_2_GaAgF_6_, and (**d**) Cs_2_GaAgF_6_ absorber based DPSCs.

Figure 11a–d displays the energy band diagrams for A_2_GaAgF_6_ based-PSCs. The VB and CB offsets, indicating the discrepancies in the VB between the absorber layer and the HTL, are dictated by each ETL within the design. The absorber layer, A_2_GaAgF_6_ (A = Na, K, Rb, and Cs), is combined with PTAA as HTL. The alignment of energy levels profoundly affects the performance and efficiency of cells^85^. In these cells, electrons and holes are introduced into the CB of the ETL and then transported to the HTL. The accumulated holes and electrons are subsequently collected at the FTO and Ni contacts, respectively. Energy band mismatches occur at the A_2_GaAgF_6_/HTL and ETL/A_2_GaAgF_6_ interfaces, significantly affecting the device’s performance. The interfacial characteristics are essential in reducing interfacial recombination. For optimal electron extraction, the electron affinity of the ETL must surpass that of the absorber at the ETL/A_2_GaAgF_6_ interface, whereas the ionization energy of the HTL should be inferior to that of the absorber at the A_2_GaAgF_6_/HTL interface to facilitate effective hole extraction. In the best designs of A_2_GaAgF_6_-based devices, Fermi level intersects the CB. This behavior is apparent in the Na_2_GaAgF_6_-based device arrangement, wherein In_2_S_3_ functions as the ETL and PTAA as HTL, as illustrated in Fig. 11a. Figure 11b–d depicts similar setups for alternative absorber materials, namely K_2_GaAgF_6_, Rb_2_GaAgF_6_, and Cs_2_GaAgF_6_, in conjunction with PTAA as HTL and In_2_S_3_ as ETL. The bandgaps of Na_2_GaAgF_6_, K_2_GaAgF_6_, Rb_2_GaAgF_6_, and Cs_2_GaAgF_6_ are 1.55, 2.203, 2.396, and 2.698 eV, respectively, and their results exhibit similarities when employing the same heterostructure. The quasi-Fermi levels, F_n_ and F_p_, are aligned with the VB energy, with F_p_ positioned above the valence band (E_V_) and F_n_ aligning with the conduction band (E_C_), so assuring consistent energy behavior across the device architecture.

### Impact of absorber thickness in inorganic A_2_GaAgF_6_ double perovskites

**Fig. 12:**
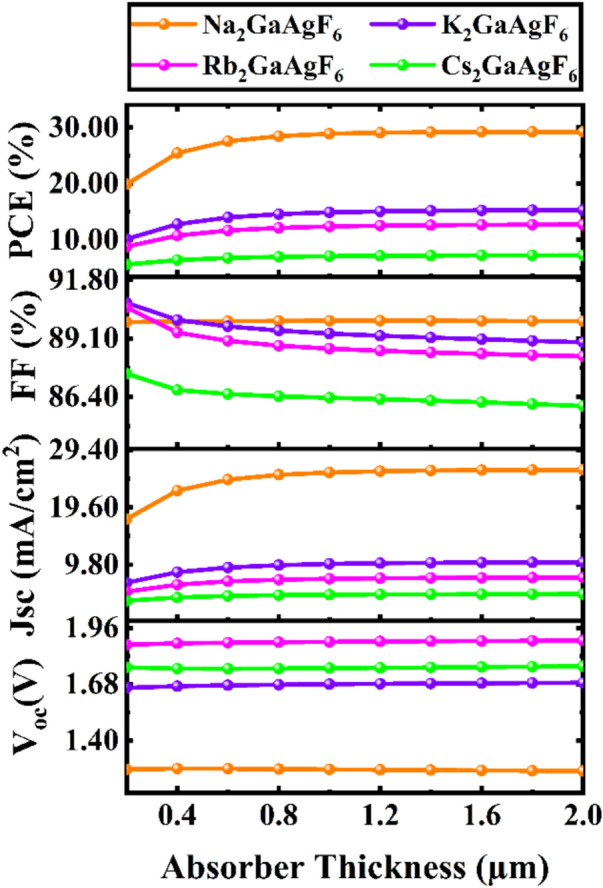
Impact of variation in A_2_GaAgF_6_-based absorber layer thickness.

Figure 12 illustrates the impact of absorber layer thickness, varying from 0.2 to 2.0 µm, on the photovoltaic properties of A_2_GaAgF_6_ (A = Na, K, Rb, Cs) thin-film solar cells. The thickness of the absorber is crucial in influencing device performance, since it directly affects light absorption efficiency, carrier production, and charge transport dynamics in the active region. Augmenting the thickness prolongs the optical path length, facilitating the absorption of a greater fraction of incident photons, especially in the longer-wavelength spectrum. This improved light-matter interaction causes a red shift in the external QE spectrum and a consistent increase in J_SC_, as additional photogenerated carriers promote current production^86^. Nonetheless, above a certain critical thickness, the advantages of enhanced absorption are negated by heightened bulk and interfacial recombination losses. Thicker layers result in extended carrier transport pathways, increasing the probability of diffusion-limited recombination prior to carriers reaching the transport layers. As a result, the J_SC_ typically reaches saturation or experiences a minor decrease, whereas the V_OC_ remains relatively constant, signifying steady electrostatic potential and efficient charge separation at the heterojunction interface. The observed variations in V_OC_ and J_SC_ across all compositions align with previously documented thickness-dependent patterns in double perovskites^87^^88^. K_2_GaAgF_6_, Rb_2_GaAgF_6_, and Cs_2_GaAgF_6_ displayed a little decline in FF with increased thickness due to heightened recombination, while Na_2_GaAgF_6_ preserved a consistent FF, indicating enhanced carrier extraction and less trap-mediated losses. The PCE of all compositions improved with thickness, reaching an optimal absorber thickness of roughly 1000 nm, which provided an ideal balance between photon absorption and reduced recombination. Beyond this threshold, further thickness yielded minimal optical benefits while markedly increasing carrier recombination, resulting in the saturation of both J_SC_ and PCE.

These results correspond with the findings of Ayyaz et al.^14^ regarding A_2_NaAlI_6_ (A = Rb, Cs) and Khan et al.^13^ concerning X_2_MgGeI_6_ (X = Rb, Cs), both of which identified an optimal absorber thickness for effective charge-carrier generation and collection in analogous double-perovskite structures. At the optimized thickness of 1000 nm, the simulated device parameters were as follows: V_OC_ values of 1.256 V (Na_2_GaAgF_6_), 1.680 V (K_2_GaAgF_6_), 1.890 V (Rb_2_GaAgF_6_), and 1.759 V (Cs_2_GaAgF_6_); J_SC_ values of 25.55 mA/cm^2^, 9.92 mA/cm^2^, 7.38 mA/cm^2^, and 4.66 mA/cm^2^; FF values of 89.92%, 89.32%, 88.63%, and 86.36%; and PCEs of 28.87%, 14.89%, 12.38%, and 7.09%, respectively. Na_2_GaAgF_6_ demonstrated superior photovoltaic performance, attaining a remarkable PCE of 28.87%, with well-balanced V_OC_, J_SC_, and FF values underscoring its potential as a high-efficiency, lead-free double perovskite absorber.

### Defect and acceptor density variations in the A_2_GaAgF_6_ absorber layer

**Fig. 13:**
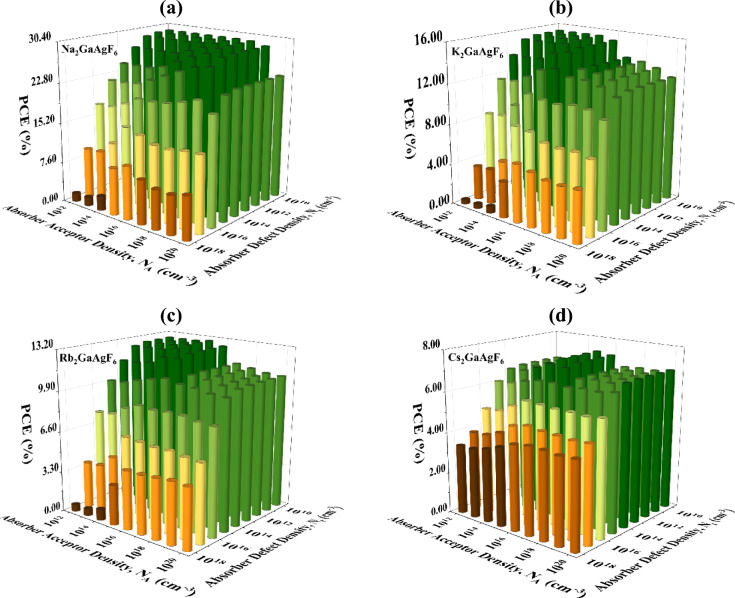
Investigation of the influence of defect and acceptor density on PCE in PSCs utilizing (**a**) Na_2_GaAgF_6_, (**b**) K_2_GaAgF_6_, (**c**) Rb_2_GaAgF_6_, and (**d**) Cs_2_GaAgF_6_ absorbers.

An optimal absorber defect density (N_t_) and acceptor density (N_A_) offered the maximum efficiency for the SCs. The quality and defect attributes of the absorber layer profoundly affect the device’s performance, as photogeneration, recombination, and charge transport transpire within the absorber^89^. The absorber layer produces photoelectrons when exposed to light. Figure 13a-d illustrates the influence of absorber N_t_ and N_A_ on PCE. The associated fluctuations in V_OC_, J_SC_, and FF are provided in the Figs. S1-S3a-d (Supplementary Information). The values of N_t_ and N_A_ were varied from 10^10^ −10^18^ cm^−3^ and from 10^12^—10^20^ cm^−3^, respectively.

Fig. S1a-d shows that for Na_2_GaAgF_6_, K_2_GaAgF_6_, Rb_2_GaAgF_6_, and Cs_2_GaAgF_6_ absorbers in the Al/FTO/In_2_S_3_/A_2_GaAgF_6_/PTAA/Ni device configuration, the V_OC_ decreases with increasing N_t_. Specifically, V_OC_ declines from 1.279 to 0.656 V, 1.761 to 1.153 V, 1.932 to 1.377 V, and 2.517 to 1.758 V for Na, K, Rb, and Cs-based compositions, respectively. The V_OC_ denotes the highest potential difference in the absence of current, reflecting the inherent potential between p-type and n-type layers. The decline in V_OC_ with rising bulk and interface defect densities is mainly attributed to increased Shockley–Read–Hall (SRH) recombination, which elevates the dark saturation current (J_0_) and diminishes the quasi-Fermi level splitting^90^. Moreover, heightened recombination at the ETL/absorber interface diminishes J_SC_, constraining carrier extraction efficiency. The negative correlation between J_0_ and V_OC_ corresponds with the relationship between J_SC_, J_0_, and V_OC_ established by Green and Cells^91^, present in Eq. (12).

12 $${V}_{OC}=\frac{nkT}{e}\left[\frac{{J}_{SC}}{{J}_{O}}+1\right]$$

In the equation, $\frac{nkT}{e}$ denotes the thermal voltage, where n is a coefficient. Fig. S2(a–d) illustrates that the J_SC_ values for the A_2_GaAgF_6_ structures vary from 25.73 to 8.57 mA/cm^2^ for Na_2_GaAgF_6_, 10.10 to 1.74 mA/cm^2^ for K_2_GaAgF_6_, 7.58 to 1.79 mA/cm^2^ for Rb_2_GaAgF_6_, and 4.81 to 2.56 mA/cm^2^ for Cs_2_GaAgF_6_. J_SC_ denotes the current generated when the terminals of a solar cell are shorted, caused by light-induced charge carriers. The value of J_SC_ is affected by the absorbed photons, the surface area of the SC, and its optical properties.

Fig. S3a–d illustrates that Na_2_GaAgF_6_ achieves a peak FF of 90.2% at absorber N_A_ ranging from 10^16^ −10^18^ cm^−3^ and N_t_ between 10^10^—10^14^ cm^−3^. The K_2_GaAgF_6_ absorber demonstrated a consistent FF of 90.6% with an N_A_ between 10^18^ and 10^20^ cm^−3^ and a N_t_ between 10^12^ and 10^15^ cm^−3^. Absorbers Rb_2_GaAgF_6_ and Cs_2_GaAgF_6_ exhibited FFs of 90.17% and 88.4%, respectively, with defect densities between 10^13^ and 10^15^ cm^−3^ and acceptor densities from 10^16^ to 10^19^ cm^−3^. The FF denotes the ratio of maximum power output to the theoretical limit (V_OC_ × J_SC_) and is affected by junction electric fields and recombination in the depletion region.

Figure 13a–d illustrates the fluctuation in PCE for A_2_GaAgF_6_-based SCs, contingent upon varying acceptor densities (N_A_, 10^12^—10^20^ cm^−3^) and defect densities (N_t_, 10^10^—10^18^ cm^−3^). The PCE, representing the proportion of incident sunlight transformed into electrical energy. The Na_2_GaAgF_6_ absorber device demonstrated the maximum efficiency of 28.87%, with a N_A_ range of 10^12^—10^20^ cm^−3^ and a N_t_ range of 10^10^—10^18^ cm^−3^. Conversely, devices utilizing K_2_GaAgF_6_ and Rb_2_GaAgF_6_ absorbers, characterized by defect levels ranging from 10^12^ to 10^15^ cm^−3^ and acceptor densities between 10^16^ and 10^18^ cm^−3^, demonstrated consistent yet inferior efficiencies of 14.89%, 12.38%, and 7.09%, respectively. The PSC utilizing Cs_2_GaAgF_6_ as the absorber exhibited the lowest efficiency at 7.09%, characterized by an N_A_ ranging from 10^16^ to 10^18^ cm^−3^ and a N_t_ about 10^14^ cm^−3^. Prior studies demonstrate that PCE rises with the width of the absorber and the thickness of the ETL until a threshold is reached^92^, beyond which additional increases in both variables yield declining returns.

### J-V and QE properties

**Fig. 14:**
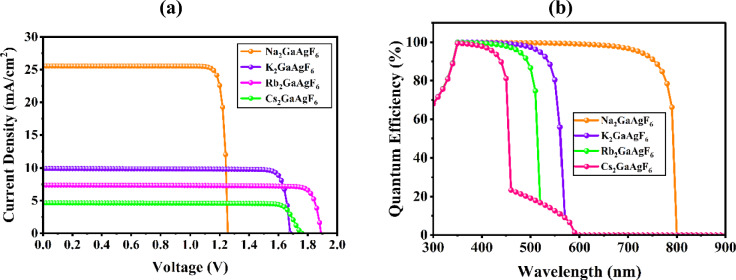
(**a**) J-V and (**b**) Q-E properties of proposed DPSCs for optimized absorber layer.

Figure 14a,b and Fig. S4a–h highlights the J-V and QE properties of A_2_GaAgF_6_-based inorganic SCs with absorber thicknesses varying from 0.2 to 2.0 µm. A_2_GaAgF_6_ demonstrates potential as a PV absorber material, with the J-V and QE metrics being markedly affected by the depth of the absorber. Thicker absorber layers can improve light absorption and photocurrent, resulting in higher J_SC_; but they may also cause increased recombination losses, which diminish V_OC_, FF, and efficiency. Fig. S4a,c,e,g illustrates the J-V characteristics for the four absorber configurations, including Na_2_GaAgF_6_, K_2_GaAgF_6_, Rb_2_GaAgF_6_, and Cs_2_GaAgF_6_, highlighting the enhanced performance of these materials. Figure 14a illustrates the optimal J-V properties for different setups, highlighting the influence of absorber thickness on SC efficacy. The ideal absorber thickness, determined by systematic investigation, was established at 1000 nm which is align with prior research^93,94^. At this ideal thickness, the V_OC_ and J_SC_ values for Na_2_GaAgF_6_, K_2_GaAgF_6_, Rb_2_GaAgF_6_, and Cs_2_GaAgF_6_ were 1.256 V and 25.55 mA/cm^2^, 1.680 V and 9.92 mA/cm^2^, 1.890 V and 7.38 mA/cm^2^, and 1.759 V and 4.66 mA/cm^2^, respectively. A thinner (1000 nm) Na_2_GaAgF_2_ absorber layer enhances efficiency to 28.87% by reducing recombination and optimizing carrier collection.

The QE of the material is considerably affected by the thickness of the A_2_GaAgF_6_ absorber layer, as both photocurrent generation and photon absorption are directly correlated with layer thickness. At extended wavelengths, a thicker absorber layer often enhances QE by capturing a greater number of photons. This results from the augmented volume for photon interaction, facilitating a greater quantity of photons to be absorbed and transformed into electrical current, therefore enhancing overall efficiency^95^. Nonetheless, an overly thick absorber layer may result in excessive photon absorption, diminishing charge carrier collecting efficiency and hence reducing quantum efficiency. Fig. S4b,d,f,h illustrate similar QE response effects across various SCs with absorber thicknesses between 200 and 2000 nm. Thicker active layers elevate cell resistance and extend the diffusion path for charge carriers, perhaps detrimentally affecting the FF. As the thickness of the layers increases beyond 1000 nm, the performance improvement that results from the thicker layers begins to diminish and ultimately reaches saturation. The optimized QE properties for the four setups are shown in Fig. 14b, demonstrating that Na_2_GaAgF_6_ consistently surpasses K_2_GaAgF_6_, Rb_2_GaAgF_6_, and Cs_2_GaAgF_6_ in quantum efficiency.

Figure 14a,b present the J-V and QE characteristics for the Al/FTO/In_2_S_3_/A_2_GaAgF_6_/PTAA SC configuration, with the following FF and PCE values: 89.92% and 28.87% for Na_2_GaAgF_6_, 89.32% and 14.89% for K_2_GaAgF_6_, 88.63% and 12.38% for Rb_2_GaAgF_6_, and 86.36% and 7.09% for Cs_2_GaAgF_6_. The Al/FTO/In_2_S_3_/Na_2_GaAgF_6_/PTAA design was chosen as the optimal device owing to its enhanced performance relative to the alternative configurations. This study highlights the promise of A_2_GaAgF_6_-based materials for high-efficiency SCs, with Na_2_GaAgF_6_ exhibiting the highest efficiency.

### Comparison of SCAPS-1D results with prior research

Table 9 provides a systematic comparison of the photovoltaic performance of the four A_2_GaAgF_6_ (A = Na, K, Rb, Cs)-based device configurations examined in this study with previously documented practical and theoretical perovskite solar cells. The comparison is conducted under both optimal SCAPS-1D settings and utilizing DFT-derived absorption coefficients, thereby offering a realistic evaluation of device performance. The Na_2_GaAgF_6_-based single-junction device demonstrates the most favorable performance among all configurations, with a maximum PCE of 28.87%, with a V_OC_ of 1.25 V, a J_SC_ of 25.55 mA/cm^2^, and an FF of 89.92% under optimal conditions. Utilizing more realistic, DFT-derived optical characteristics, the device achieves a competitive performance with a PCE of 4.85%, V_OC_ of 1.11 V, J_SC_ of 4.96 mA/cm^2^, and FF of 87.80%, surpassing most previously documented double perovskite-based structures. The exceptional performance of Na_2_GaAgF_6_ is due to its diminished band gap, improved absorption in the visible spectrum, and advantageous band alignment with the chosen transport layers, which together facilitate effective photogeneration and extraction of charge carriers while inhibiting interfacial recombination. Conversely, devices utilizing K_2_GaAgF_6_, Rb_2_GaAgF_6_, and Cs_2_GaAgF_6_ have progressively diminished efficiencies, with optimal PCEs of 14.89%, 12.38%, and 7.09%, respectively, which further decline to 3.97%, 3.52%, and 2.85% with the application of DFT-derived absorption coefficients. The decline in performance is associated with the expansion of the band gap and the resultant decrease in low-energy photon absorption as the A-site ionic radius transitions from Na to Cs. In contrast to previous theoretical analyses, the current study utilizes optimized settings for the absorber and transport layers, along with realistic optical inputs, facilitating a more accurate assessment of device performance. The findings indicate that A_2_GaAgF_6_ absorbers, especially Na_2_GaAgF_6_, demonstrate enhanced optoelectronic properties and device compatibility compared to numerous previously examined double perovskites, underscoring their significant potential for the advancement of efficient, lead-free perovskite solar cells.

**Table 9:** Comparative study of double perovskite absorber layers (E = Experimental, T = Theoretical, * = This work).

Types	Structures	Absorption coefficient	PCE (%)	V_OC_ (V)	J_SC_ (mA/cm^2^)	FF (%)	Ref
E	FTO/TiO_2_/Cs_2_AgBiBr_6_/spiro-OMeTAD/Au	Experimental	2.43	0.98	3.96	62.40	^96^
E	FTO/TiO_2_/Cs_2_AgBiBr_6_/spiroOMeTAD/MoO_3_/Ag	Experimental	2.51	1.01	3.82	65	^21^
T	FTO/CdS/Ba_2_AgBiBr_6_/Cu_2_O/Au	Ideal	13.93	0.83	20.22	82.24	^41^
T	ITO/CeO_2_/Cs_2_BiAgI_6_/CBTS/Au	Ideal	14.44	0.92	23.59	66.21	^97^
T	TiO_2_/Cs_2_CuBiCl_6_/Cu_2_O	Ideal	17.03	0.91	21.66	85.99	^98^
T	Au/p-CdTe/Cs_2_TiI_6_/n-TiO_2_/ITO	Ideal	15.06	1.39	25.08	43.17	^78^
T	ITO/ZnO/Cs_2_AuBiCl_6_/CuSbS_2_	Ideal	21.16	0.68	39.94	77.72	^99^
T	TCO/IDL_1_/Cs_2_AuBiCl_6_/IDL_2_/Cu_2_O	Ideal	22.18	0.86	31.69	81.17	^100^
T	FTO/TiO_2_/Cs_2_InBiBr_6_/Cu_2_O/Au	Ideal	23.64	0.79	34.14	86.72	^88^
T	FTO/SnO_2_/Cs_2_AgBiBr_6_/CuSCN/Au	Ideal	11.51	-	-	-	^101^
T	ITO/ZnO/CsSnI_3_/Spiro-OMeTAD/Ag	Ideal	26.40	-	-	-	^102^
T	Al/FTO/SnS_2_/Sr_3_SbCl_3_/V_2_O_5_/Ni	Ideal	17.52	1.39	14.09	89.01	^103^
		DFT-derived	1.12	1.27	1.12	89.93	
T	Al/FTO/PCBM/Sr_3_SbCl_3_/V_2_O_5_/Ni	Ideal	14.83	1.35	13.97	78.39	
		DFT-derived	4.43	1.35	3.61	90.38	
T	Al/FTO/SnS_2_/Li_2_PdCl_6_/Ni	Ideal	22.06	0.78	38.11	76.97	^104^
		DFT-derived	1.71	0.63	3.28	73.45	
T	Al/FTO/SnS_2_/Na_2_PdCl_6_/Ni	Ideal	25.55	0.75	42.54	79.16	
		DFT-derived	1.96	0.62	3.41	74.21	
T	Al/FTO/In_2_S_3_/Na_2_GaAgF_6_/PTAA/Ni	Ideal	28.87	1.25	25.55	89.92	*
		DFT-derived	4.86	1.11	4.96	87.80	
T	Al/FTO/In_2_S_3_/K_2_GaAgF_6_/PTAA/Ni	Ideal	14.89	1.68	9.92	89.32	*
		DFT-derived	3.97	1.46	3.08	88.26	
T	Al/FTO/In_2_S_3_/Rb_2_GaAgF_6_/PTAA/Ni	Ideal	12.38	1.89	7.38	88.63	*
		DFT-derived	3.52	1.69	2.55	81.43	
T	Al/FTO/In_2_S_3_/Cs_2_GaAgF_6_/PTAA/Ni	Ideal	7.09	1.75	4.66	86.36	*
		DFT-derived	2.85	1.55	1.64	80.65	

## Machine learning models

A machine learning (ML)-assisted analysis was conducted utilizing the SCAPS-1D simulation dataset to enhance predicted accuracy and expedite device performance modification. In recent years, machine learning approaches have emerged as potent instruments in photovoltaic research, facilitating the effective modeling of intricate and nonlinear interactions among material properties, device parameters, and solar cell efficiency. Numerous studies have evidenced the effective amalgamation of machine learning with SCAPS simulations to accurately anticipate and enhance the performance of PSC^32^^,^^105^. The Random Forest Regressor (RFR) has garnered significant attention among many machine learning algorithms because of its robustness, superior predictive capability, and resilience to overfitting. Singh et al.^106^ utilized Random Forest models to enhance transport layer characteristics in perovskite solar cells, demonstrating significant concordance with SCAPS simulation outcomes. Hossain et al.^107^ and Reza et al.^90^ similarly proved that Random Forest-based models can accurately predict photovoltaic performance while effectively identifying critical regulating parameters. This research collectively affirms that RFR is especially adept for PV applications owing to its capacity to describe nonlinear interactions and deliver interpretable feature significance. Inspired by these achievements, this study utilizes a Random Forest Regressor inside a hybrid SCAPS-ML framework to build a predictive correlation between input device parameters and PCE. In contrast to traditional SCAPS-1D simulations that depend on the sequential numerical resolution of semiconductor transport equations, the RFR model permits the concurrent examination of numerous interacting variables and allows for the swift identification of concealed correlations within the dataset. The training dataset was produced using comprehensive SCAPS-1D simulations encompassing a broad spectrum of device setups and input parameters. This machine learning-assisted method improves predictive efficiency and facilitates the identification of key performance-driving factors via feature importance analysis. The amalgamation of Random Forest modeling with SCAPS simulations yields a computationally efficient, precise, and physically interpretable framework for the design and optimization of high-performance, lead-free double perovskite solar cells.

### Data processing and dataset verification

#### Dataset creation and overview

A detailed dataset was created utilizing SCAPS-1D (version 3309) simulations to facilitate machine learning study of perovskite solar cell performance. The dataset was created by methodically altering essential physical and operational parameters that influence photovoltaic performance. All simulation outputs were handled in a Python environment utilizing the Pandas and NumPy modules for efficient data management, organizing, and preprocessing^108^. A total of 1,600 device configurations were simulated, each defined by 12 input features, including absorber thickness, bandgap, defect density, doping concentration, and interfacial parameters. This produced a dataset of 19,200 (1600 device × 12 input features) data points, divided into 15,360 training samples (80%) and 3,840 testing samples (20%). The dataset has a broad spectrum of values, with specific features displaying considerable fluctuation. The shallow acceptor density of Na_2_GaAgF_6_ spans from 1.18 × 10^13^ to 1.0 × 10^20^ cm^−3^, with efficiency fluctuating between 14.705% and 31.906%. The data samples are used to make predictions and analyses performance, specifically aimed at enhancing the efficacy of PSCs^109^. Table 10 offers a detailed summary of the dataset statistics, showcasing the ranges and statistical metrics of the key features. It distinctly illustrates the distribution of variables and their corresponding ranges. The dataset reveals that major performance measures contribute variably to the total efficiency of SCs. The mean V_OC_ is 1.256 V, J_SC_ is 25.55 mA/cm^2^, FF is 89.92%, and efficiency is 28.87%. The values indicate that the dataset reflects moderate performance for voltage and current generation. The efficiency fluctuates based on material qualities, such as absorber band gap and buffer layer attributes. The cumulative impact of these characteristics ultimately determines the total efficiency of the PSC. Enhancing the V_OC_, J_SC_, and FF is crucial for enhancing the device’s efficiency.

**Table 10:** Summary for 12 features dataset.

Variables	Points	Std. deviation	Min—max
Absorber thickness	100	0.0101	0.2—1.2
Acceptor density		–	1.18 × 10^13^—1 × 10^20^
Defect density		–	1 × 10^10^ – 7.22 × 10^16^
Band gap		0.00404	1.3–1.7
Electron affinity		0.00606	3.7–4.3
CB effective DOS		–	1 × 10^18^ – 9.45 × 10^20^
VB effective DOS		–	1 × 10^17^ – 1 × 10^20^
HTL thickness		0.00758	0.05–0.8
ETL thickness		0.00758	0.05–0.8
ETL doping density		–	1.18 × 10^13^—1 × 10^20^
HTL doping density		–	1.18 × 10^13^—1 × 10^20^
Interface defect		–	1 × 10^09^—1 × 10^16^

#### Data processing and feature engineering

The data processing phase was executed to guarantee dataset consistency and appropriateness for machine learning applications. A key problem in multi-parameter systems is the disparity in numerical scales among various physical variables. Feature normalization was implemented to standardize all variables on a uniform numerical scale, enhancing computational stability and model efficacy. An additional improvement entails feature engineering for performance classification. A categorical variable was created to classify device performance into three distinct groups: “Good,” “Worst,” and “Extremely Worst” for improved interpretation of efficiency trends. This classification was established based on the statistical distribution of efficiency values utilizing the mean (μ) and standard deviation (σ), articulated in Eq. (13)^110^.

13 $$T= \left[\sigma -std,\sigma ,\sigma +std\right]$$

This statistical thresholding facilitated the methodical categorization of device performance regimes, enhancing the interpretability of efficiency variances across diverse configurations.

#### Data examination and distribution assessment

**Fig. 15:**
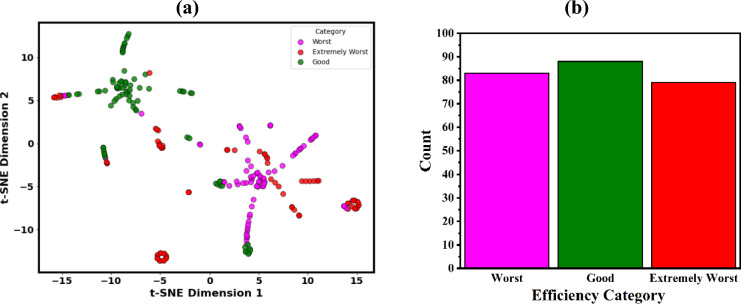
(**a**) T-SNE visualizations and (**b**) different class proportions for datasets.

A comprehensive exploration data analysis (EDA) was conducted to evaluate data quality, identify discrepancies, and comprehend underlying feature relationships. Statistical summaries, histograms, and correlation matrices were utilized to detect outliers, missing values, and non-physical data points. Unrealistic entries, including negative thickness values or physically implausible defect densities, were meticulously eliminated to maintain dataset integrity. The t-distributed Stochastic Neighbor Embedding (t-SNE) technique was utilized to illustrate the high-dimensional structure of the dataset^111^, demonstrated in Fig. 15a. The resultant two-dimensional projection demonstrated considerable overlap among efficiency classes, signifying robust nonlinear correlations between input variables and output performance. This overlap underscores the inherent complexity of the dataset and the challenge of defining distinct decision boundaries in a diminished feature space. Figure 15b illustrates the class distribution was somewhat balanced, comprising 88 "Good," 83 "Worst," and 79 “Extremely Worst” samples. This equilibrium mitigates sampling bias in model training, although it simultaneously escalates classification complexity owing to overlapping feature distributions. This underscores the necessity for resilient machine learning models adept at managing nonlinear and high-dimensional interactions.

#### Feature normalization and dataset integrity

Despite tree-based models like Random Forest being typically unaffected by feature scaling, standardization was implemented to achieve consistency across variables with varying physical units and magnitudes. The normalization process was meticulously executed to prevent data leaking by applying transformations exclusively to the training subset. After preprocessing, the dataset was subjected to stringent validation, encompassing dimensional consistency verification, statistical reevaluation, and correlation examination. The resulting dataset was verified as complete, devoid of missing values, and physically consistent. This curated dataset offers a solid basis for future predictive modeling and performance evaluation.

### Model validation approach

#### Data partitioning and training protocol

To assess the predicted efficacy of the machine learning model, the dataset was partitioned into training and testing sets with an 80:20 split ratio. The training dataset (80%) was utilized to construct the Random Forest Regressor (RFR) model, and the remaining 20% was allocated for independent evaluation. This method guarantees an impartial assessment of model generalization on novel data.

#### Metrics for performance evaluation

The efficacy of the RFR model was evaluated by many statistical metrics, including the coefficient of determination (R^2^), mean squared error (MSE), mean absolute error (MAE), and root mean square error (RMSE). These indicators combined offer a thorough assessment of predictive accuracy, error magnitude, and model stability. Alongside numerical measures, graphical evaluation methods like projected vs real plots, error distribution histograms, and correlation heatmaps were utilized. These visual instruments further validate the concordance between SCAPS-generated values and ML-predicted outputs, while also emphasizing any minor discrepancies or biases.

#### Validation methodology and model resilience

This work employed a hold-out validation technique owing to the moderate sample size and considerations of computing efficiency^112^. Although k-fold cross-validation is frequently favored for its statistical robustness, the selected method offers an equitable compromise between computational expense and dependable performance assessment^113^. The intrinsic architecture of the Random Forest algorithm, founded on bootstrap aggregation (bagging) and ensemble learning, bolsters model resilience by diminishing variance and alleviating overfitting. This facilitates robust and dependable predictions across many device configurations.

#### Comprehensive validation framework

The validation framework integrates quantitative performance measurements with qualitative visualization methods, facilitating thorough assessment of model behavior. This integrated method demonstrates that the created model possesses robust predictive accuracy, significant generalization ability, and consistent learning behavior. The proposed SCAPS–ML framework offers a computationally efficient and physiologically interpretable method for predicting solar performance. It markedly decreases computational demands relative to traditional modeling techniques, facilitating swift evaluation and enhancement of high-efficiency, lead-free double perovskite solar cells.

### Feature importance analysis

**Fig. 16:**
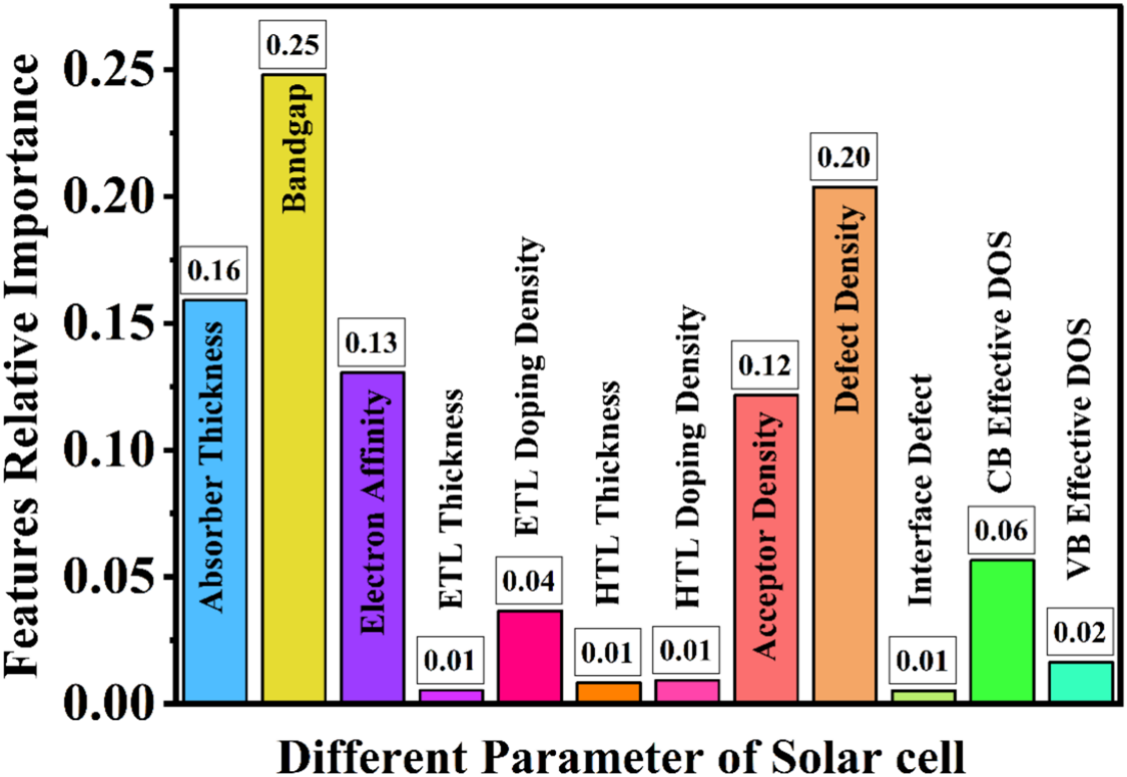
Impact of individuals is attributed to the predictions of the ML model.

The Scikit-Learn package was utilized to assess the impact of each feature on the overall efficacy of the ML model. The twelve most significant features were selected, and their relative value was depicted on Fig. 16. Key parameters, including band gap, absorber thickness, and defect density, were identified as the principal factors determining the performance of PSCs. These findings offer significant insights for the development of novel, more efficient SCs. The chosen key elements were then used to create a new model that aimed to achieve comparable performance while focusing on similar features and model size. The outcomes of this optimized model were presented in Fig. S5a-d. Ideally, the slope of the plots should approximate 1, signifying precise predictions^114^. The ML model attained a mean accuracy of 97.2%, with R^2^ values of 0.9726 for PCE, 0.9767 for V_OC_, 0.9801 for J_SC_, and 0.9625 for FF in the comparison of actual and predicted values for each target feature. While the accuracy may not be exceptionally high, the results are encouraging and indicate possibilities for further investigation, especially in connecting ML and materials science. The model, trained on extensive input data, exhibited great efficiency and generated realistic outcomes, making it a significant asset for future research and optimization of SC design.

### Correlation heatmap and SHAP analysis of proposed cell

**Fig. 17:**
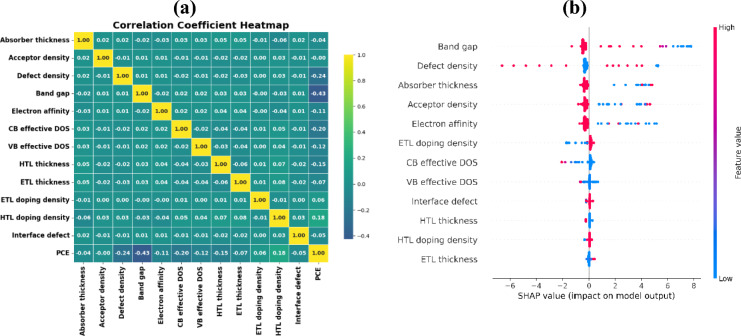
(**a**) Correlation heatmap and (**b**) SHAP analysis on model outputs.

Figure 17a displays a heatmap that depicts the correlation coefficients across several factors in a PV materials dataset. This heatmap illustrates the linear correlations among multiple parameters. The correlation between absorber thickness and PCE is −0.43, indicating a moderate inverse relationship. The correlation between electron affinity and band gap is 0.02, signifying a very negligible connection between these two variables. The heatmap also emphasizes notable relationships across other parameters, including HTL thickness, ETL thickness, and doping densities, with certain interactions being stronger than others. This heatmap serves as a crucial tool for understanding the interrelations among diverse material qualities and their possible impact on the performance of solar systems, especially concerning efficiency.

Figure 17b illustrates the SHAP (Shapley Additive Explanations)-based assessment of feature impact on the model. The SHAP plot is a necessary tool for identifying the physical properties of factors that most profoundly affect the target variable^115^. The graphic clearly demonstrates that defect level has the most significant negative impact on the model’s predictions. On the other hand, transport layer properties including thickness, doping levels, have minimal impact on SC performance. The identification indicates that an increase in defect level results in a decrease in the anticipated target value.

### Performance evaluation of random forest models

**Fig. 18:**
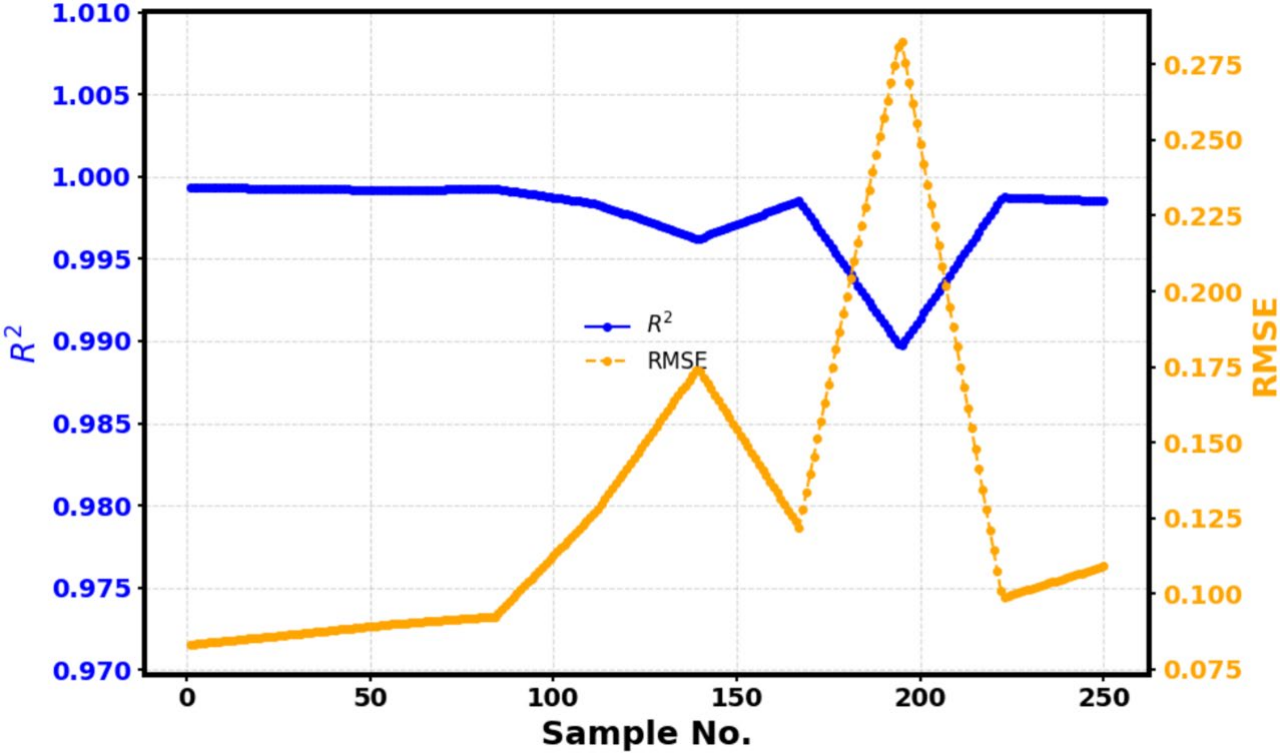
Performance of ML models using J-V curve prediction.

A thorough examination of the RF regression technique was performed to predict the performance metrics of SCs. The forecasting capability of this ML model, especially for critical performance parameters, is illustrated in Table 11. The measures used to evaluate model performance comprise MSE, MAE, RMSE, and R^2^. Each indicator provides a unique insight into the model’s accuracy, error magnitude, and generalizability, collectively providing a thorough assessment of the model’s predictive capability. The RF model reliably attains minimal RMSE values for all target parameters. Conversely, elevated R^2^ values remain high across all targets, confirming a strong connection between the estimated and real values. This underscores RF as a extremely efficient model for predicting essential SC properties. The J-V curve is an essential instrument, offering insights into device behavior, performance evaluation, and design enhancement. It illustrates the nonlinear properties of diodes and aids in determining essential values. A total of 250 randomly chosen samples were utilized to forecast the J-V curve. The RMSE and R^2^ data illustrated in Fig. 18 exhibit elevated R^2^ values averaging 0.972 and low RMSE values averaging 0.177 between the actual and projected curves. The negligible deviations underscore the accuracy of the fitted curve closely aligning with the real data, thereby facilitating dependable prediction of the J-V curve.

**Table 11:** Regression-based evaluation of photovoltaic parameter prediction.

Parameters	R^2^	MAE	MSE	RMSE
V_OC_	0.9767	0.0216	0.0823	0.1257
J_SC_	0.9801	0.0617	0.0737	0.1014
FF	0.9625	0.1014	0.1838	0.3287
PCE	0.9726	0.0434	0.0243	0.1559

## Conclusion

This study used first-principles calculations based on DFT to meticulously investigate the structural, electronic, optical, and mechanical properties of double perovskite combinations A_2_GaAgF_6_ (A = Na, K, Rb, and Cs). The lattice characteristics of the A_2_GaAgF_6_ compounds align remarkably well with theoretical results, confirming the precision of our computations. The electronic study reveals that A_2_GaAgF_6_ compounds are semiconductors with direct band gaps, as measured by the GGA-PBE function: 1.55 eV for Na_2_GaAgF_6_, 2.203 eV for K_2_GaAgF_6_, 2.396 eV for Rb_2_GaAgF_6_, and 2.698 eV for Cs_2_GaAgF_6_. An extensive optical investigation demonstrated that A_2_GaAgF_6_ compounds have robust optical properties, marked by excellent absorption, reflectivity, and energy loss functions, making them extremely suitable for optoelectronic applications. Of the four device architectures examined, the Al/FTO/In_2_S_3_/Na_2_GaAgF_6_/PTAA/Ni design exhibited superior performance, achieving a V_OC_ of 1.2563 V, J_SC_ of 25.556 mA/cm^2^, FF of 89.92%, and a PCE of 28.87%. The study assessed the influence of contacts materials, band alignment, absorber thickness, defect density, doping levels, QE, and J-V characteristics. Additionally, a machine learning methodology was utilized to ascertain optimal design parameters, exhibiting robust predictive efficacy. The Random Forest model precisely predicted key performance parameters, yielding R^2^ of 0.972 and RMSE of 0.177, demonstrating that machine learning integration can diminish development time and costs while improving device optimization and photovoltaic efficiency. Despite the encouraging simulated results, various obstacles impede the attainment of similar experimental efficiencies in A_2_GaAgF_6_-based fluoride double perovskites. Their elevated lattice ionicity affects thin-film production and the regulation of phase purity, rendering synthesis difficult. Defect development and vacancies create deep trap states, diminishing carrier mobility and lifetime, while interfacial defects, including grain boundary recombination and contact resistance, further impair charge extraction efficiency. The reported high efficiencies are predicated on idealized, low-defect models that typically reflect actual device conditions. Future investigations should focus on experimental synthesis and structural characterization, augmented by hybrid DFT methodologies (e.g., HSE06, GW) to enhance the precision of band gap predictions. Machine learning models require refinement using experimental datasets to improve predicted accuracy. Mitigating these effects via defect passivation, interface optimization, and enhanced manufacturing control will be essential to reconcile the disparity between theoretical and practical performance in lead-free fluoride perovskite solar cells.

## Supplementary Information

Supplementary Information.

## Data Availability

The data supporting the findings of this study are available from the corresponding authors upon reasonable request. Correspondence and requests for materials should be addressed to Mekuria Tsegaye Alemu and Asadul Islam Shimul.
