# Supplementary material for: First principles and machine learning investigation of structural stability and optoelectronic behavior in A2GaAgF6 (A = Na, K, Rb, Cs) double perovskite solar cells
Source: Sci Rep. 2026 Apr 21;16:18576. doi: 10.1038/s41598-026-49631-8 (PMC13270016; doi:10.1038/s41598-026-49631-8)
Supplement: Supplementary file 1 — Supplementary Information. [file 41598_2026_49631_MOESM1_ESM.docx]

**First Principles and Machine Learning Investigation of Structural Stability and Optoelectronic Behavior in A_2_GaAgF_6_ (A = Na, K, Rb, Cs) Double Perovskite Solar Cells**

Asadul Islam Shimul^1***^, Karim KRIAA^2^, Bipul Chandra Biswas^1^, Chemseddine Maatki^2**^, Md. Azizur Rahman^3^, Mekuria Tsegaye Alemu^4*^, Noureddine Elboughdiri^5^

*^1^Department of Electrical and Electronic Engineering,* *Gopalganj Science and Technology University, Gopalganj-8105, Bangladesh.*

*^2^College of Engineering, Imam Mohammad Ibn Saud Islamic University (IMSIU), Riyadh, 11432, Saudi Arabia.*

*^3^Department of Electrical and Electronic Engineering, Begum Rokeya University, Rangpur 5400, Bangladesh.*

*^4^Department of Physics, College of Natural and Computational Science, Kebri Dehar University, P.O. Box 250, Kebri Dehar, Ethiopia.*

*^5^Chemical Engineering Department, College of Engineering, University of Ha’il, P.O. Box 2440, Ha'il 81441, Saudi Arabia.*

Correspondence: *mekuria.tsegaye20@uok.edu.et (Mekuria Tsegaye Alemu)

[**casmaatki@imamu.edu.sa](mailto:**casmaatki@imamu.edu.sa) (Chemseddine Maatki)

[***shimul7246@gmail.com](mailto:***shimul7246@gmail.com) (Asadul Islam Shimul)

**Table S1.** The variation of bond length within the atom of A_2_GaAgF_6_ (where A= Na, K, Rb, Cs) Perovskites.

| **Compounds** | **Bonds** | **Bond Length, L (Å)** |
| --- | --- | --- |
| **Na_2_GaAgF_6_** | Ag - F | 2.366 |
|  | Ga - F | 1.913 |
|  | Na - F | 3.034 |
|  | Na - Ga | 3.706 |
|  | Na - Ag | 3.706 |
|  | Ag - Ga | 4.280 |
| **K_2_GaAgF_6_** | Ag - F | 2.415 |
|  | Ga - F | 1.924 |
|  | K - F | 3.078 |
|  | K - Ga | 3.758 |
|  | K - Ag | 3.758 |
|  | Ag - Ga | 4.340 |
| **Rb_2_GaAgF_6_** | Ag - F | 2.461 |
|  | Ga - F | 1.933 |
|  | Rb - F | 3.118 |
|  | Rb - Ga | 3.806 |
|  | Rb - Ag | 3.806 |
|  | Ag - Ga | 4.395 |
| **Cs_2_GaAgF_6_** | Ag - F | 2.536 |
|  | Ga - F | 1.943 |
|  | Cs - F | 3.181 |
|  | Cs - Ga | 3.879 |
|  | Cs - Ag | 3.879 |
|  | Ag - Ga | 4.480 |

| **(a)** | **(b)** |
| --- | --- |
| **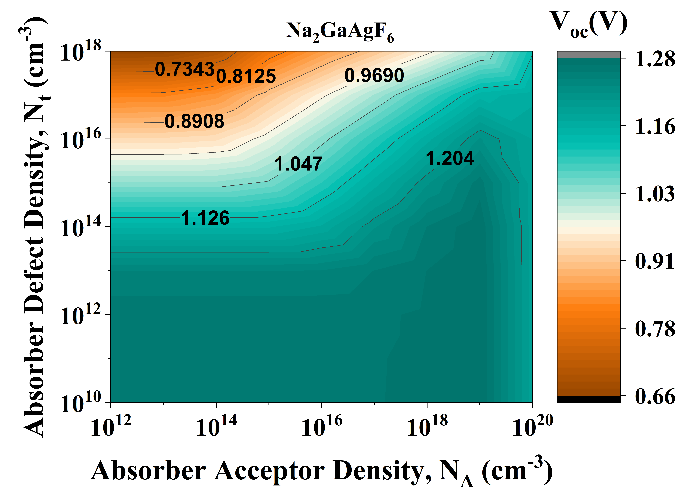** | **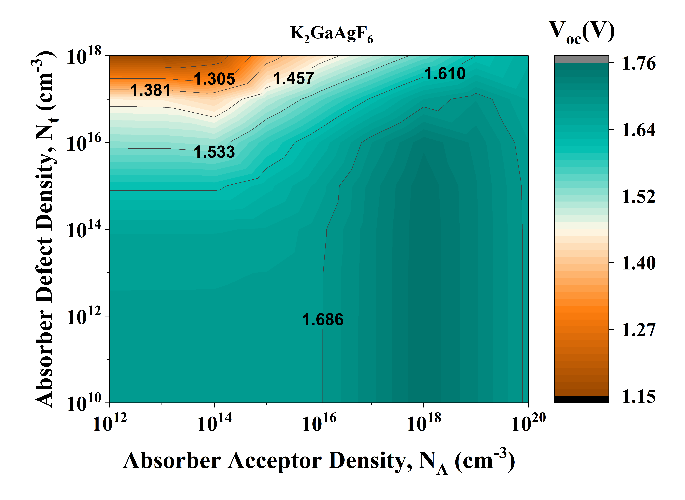** |
| **(c)** | **(d)** |
| **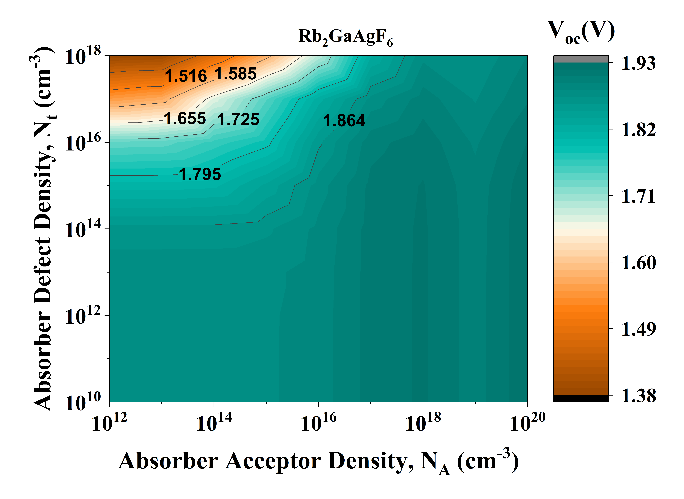** | **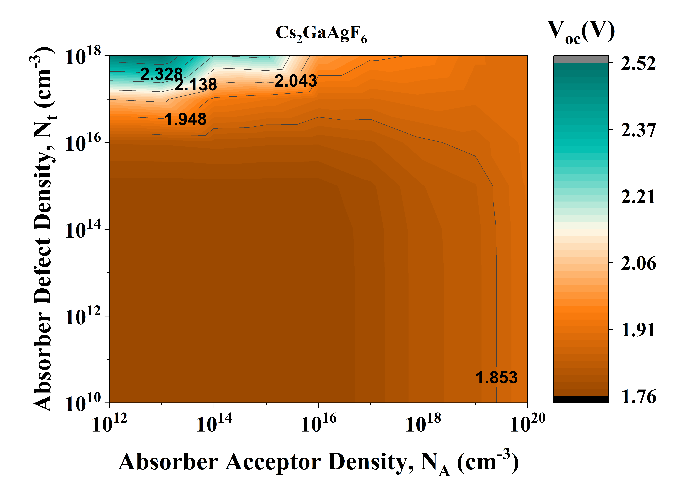** |

**Fig. S1.** Investigation of the influence of defect and acceptor density on V_OC_ in PSCs utilizing (a) Na_2_GaAgF_6_, (b) K_2_GaAgF_6_, (c) Rb_2_GaAgF_6_, and (d) Cs_2_GaAgF_6_ absorbers.

| **(a)** | **(b)** |
| --- | --- |
| **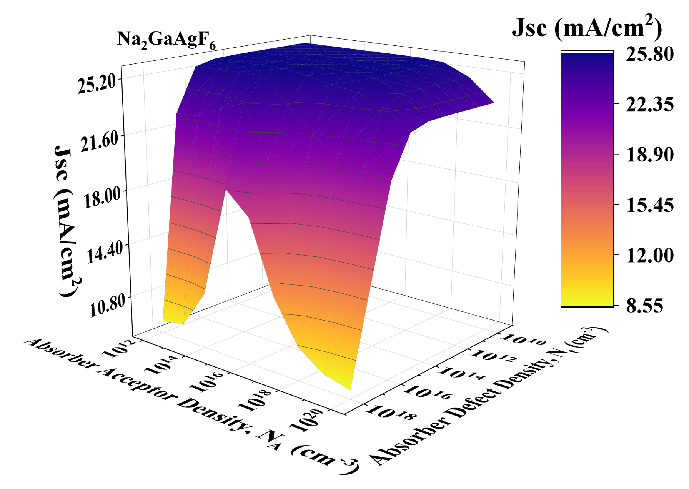** | **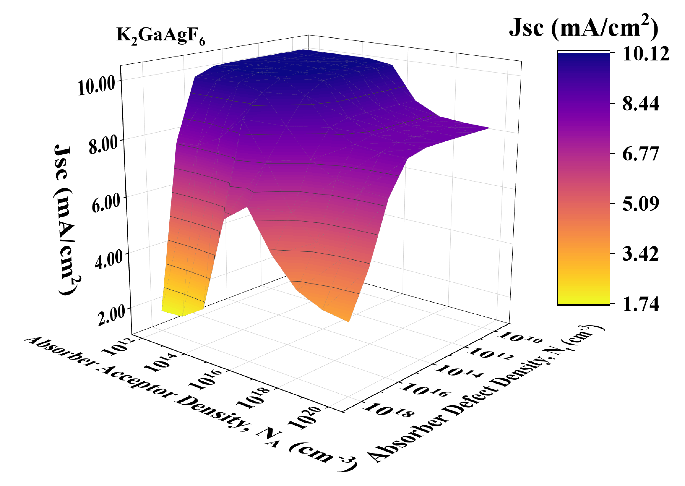** |
| **(c)** | **(d)** |
| **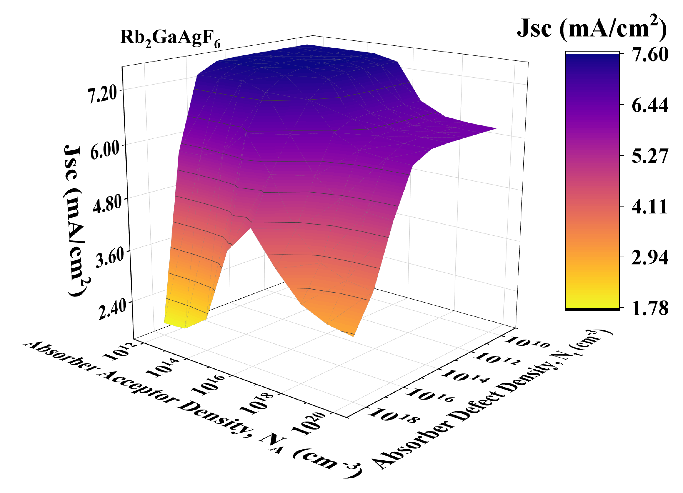** | **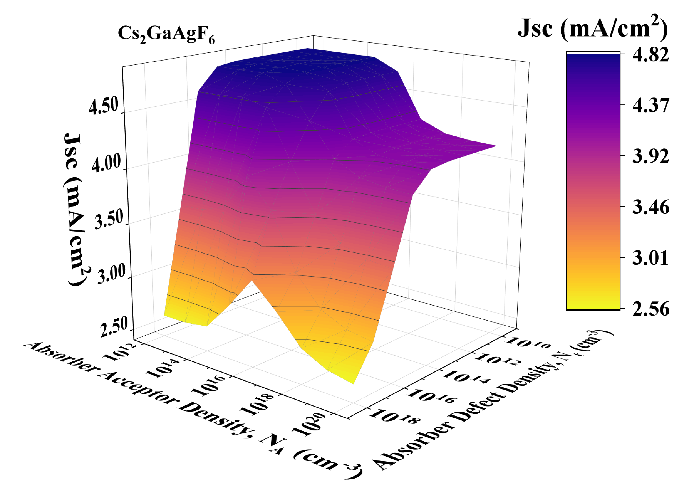** |

**Fig. S2.** Investigation of the influence of defect and acceptor density on J_SC_ in PSCs utilizing (a) Na_2_GaAgF_6_, (b) K_2_GaAgF_6_, (c) Rb_2_GaAgF_6_, and (d) Cs_2_GaAgF_6_ absorbers.

| **(a)** | **(b)** |
| --- | --- |
| **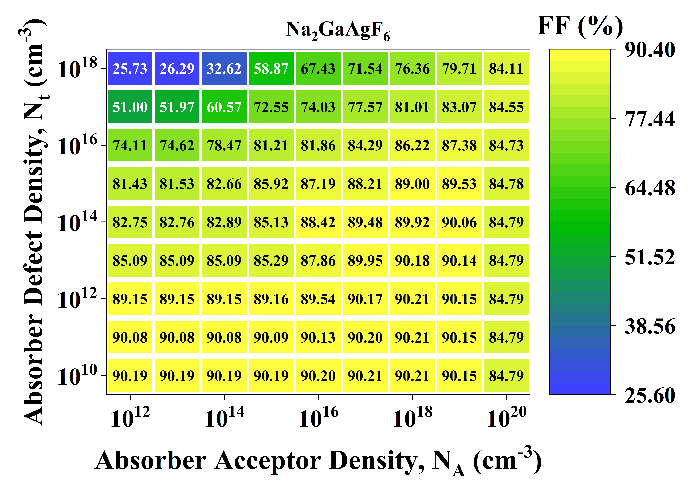** | **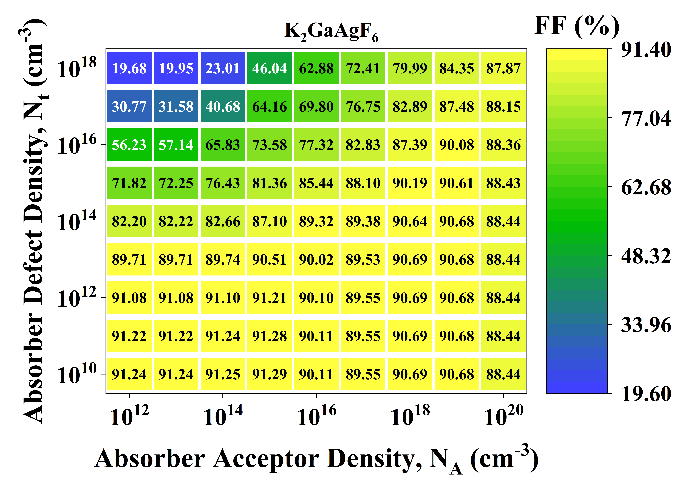** |
| **(c)** | **(d)** |
| **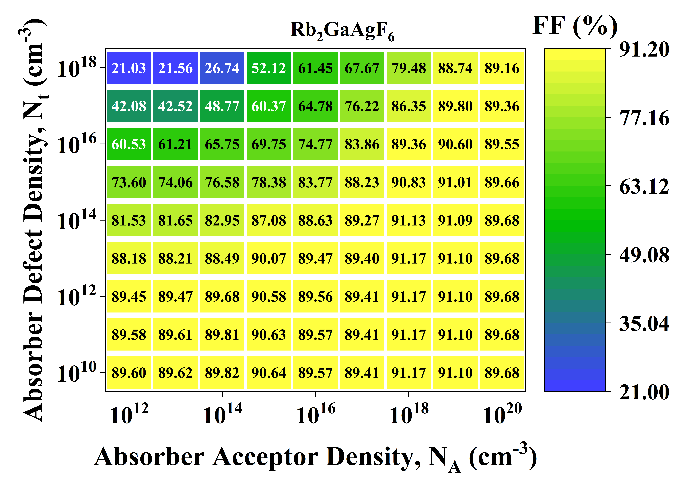** | **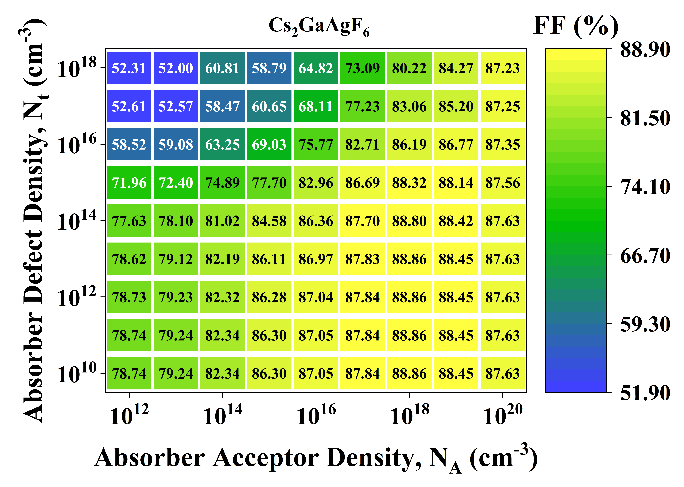** |

**Fig. S3**. Investigation of the influence of defect and acceptor density on FF in PSCs utilizing (a) Na_2_GaAgF_6_, (b) K_2_GaAgF_6_, (c) Rb_2_GaAgF_6_, and (d) Cs_2_GaAgF_6_ absorbers.

| **(a)** | **(b)** |
| --- | --- |
| **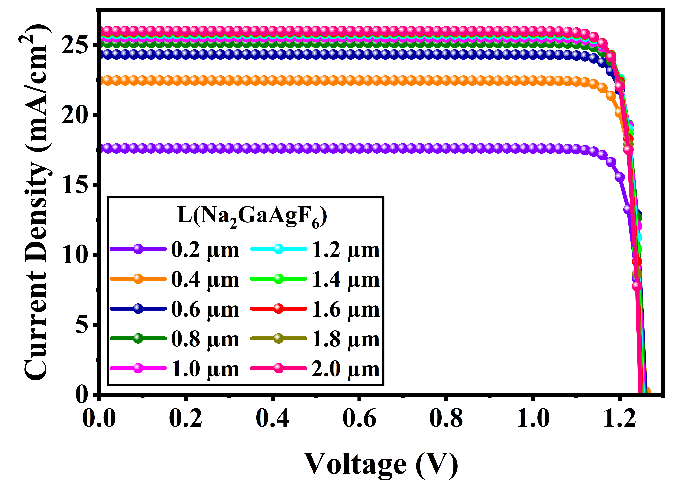** | **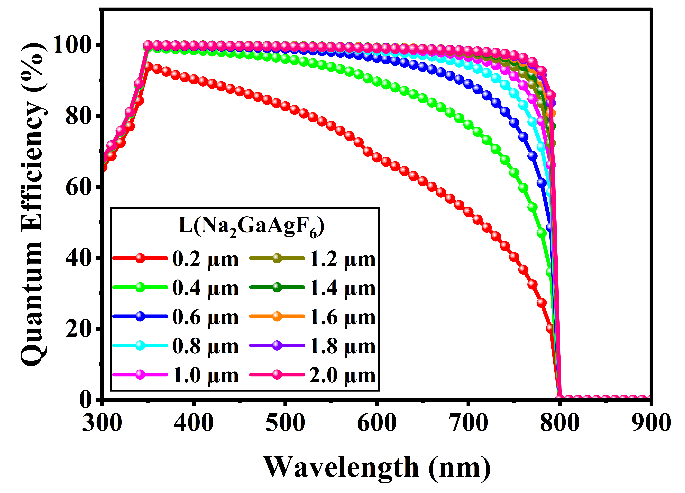** |
| **(c)** | **(d)** |
| **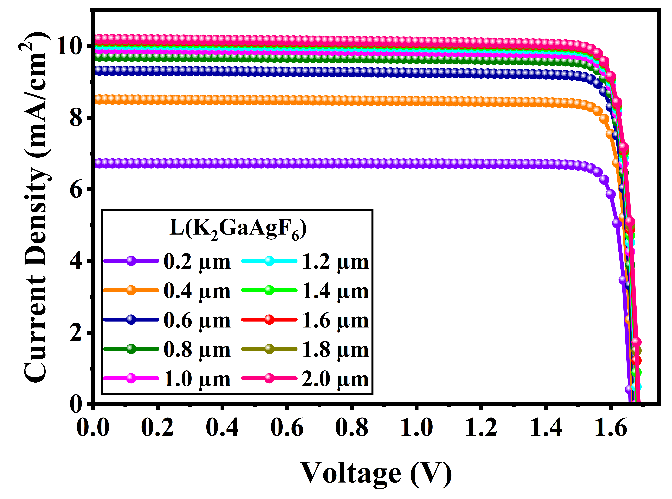** | **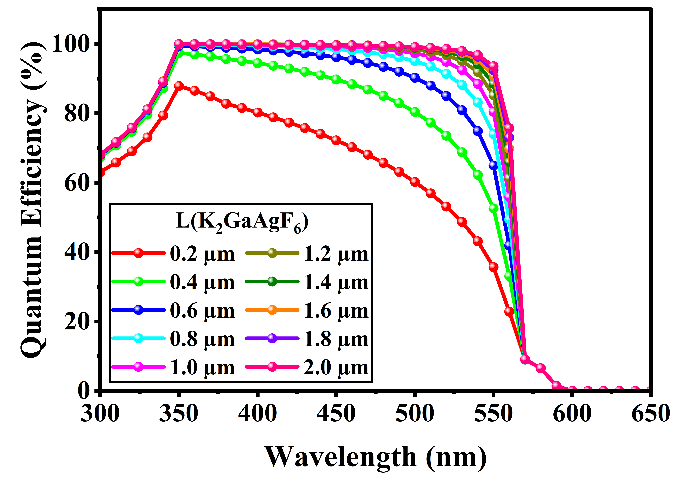** |
| **(e)** | **(f)** |
| **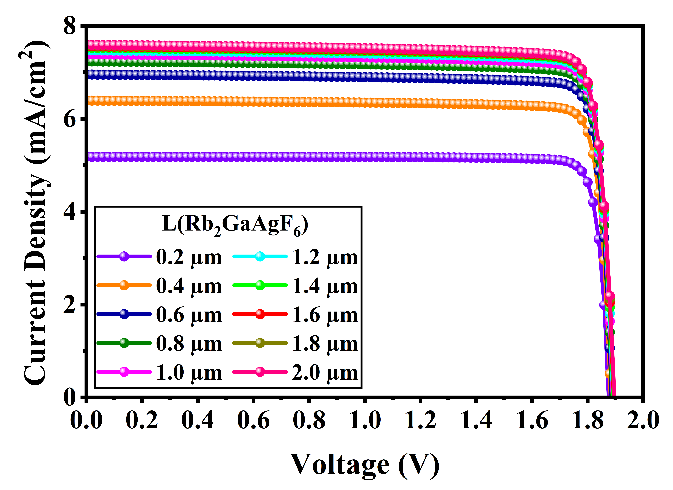** | **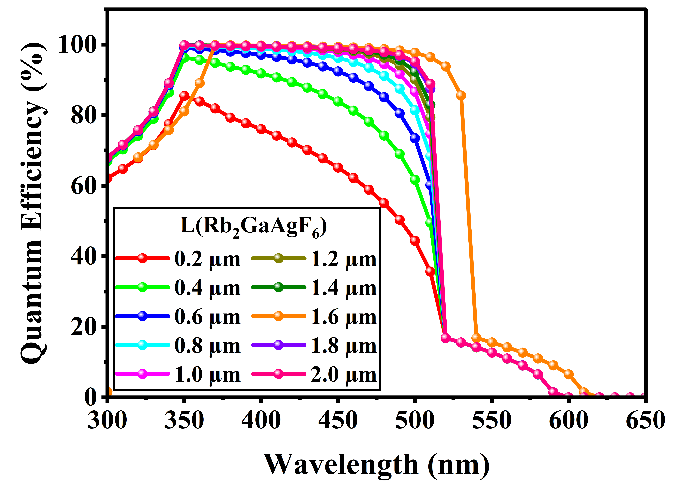** |
| **(g)** | **(h)** |
| **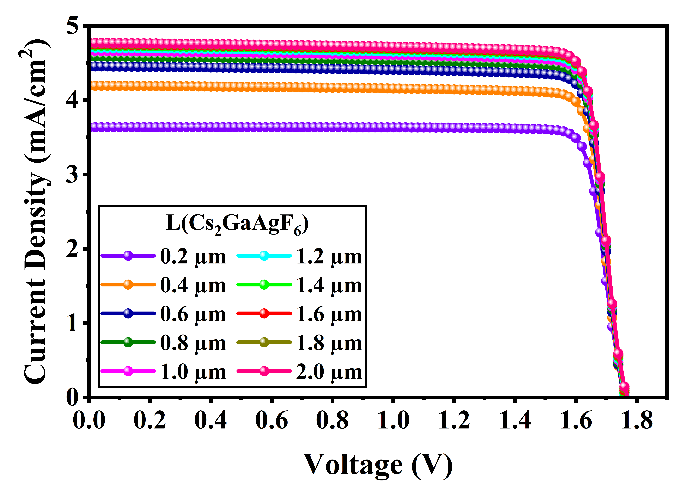** | **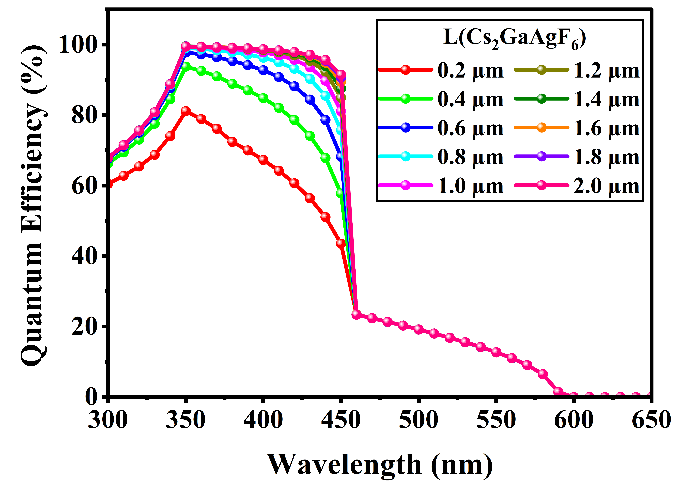** |

**Fig. S4.** J-V and Q-E properties of proposed DPSCs for (a, b) Na_2_GaAgF_6_, (c, d) K_2_GaAgF_6_, (e, f) Rb_2_GaAgF_6_, and (g, h) Cs_2_GaAgF_6_.

| **(a)** | **(b)** |
| --- | --- |
| **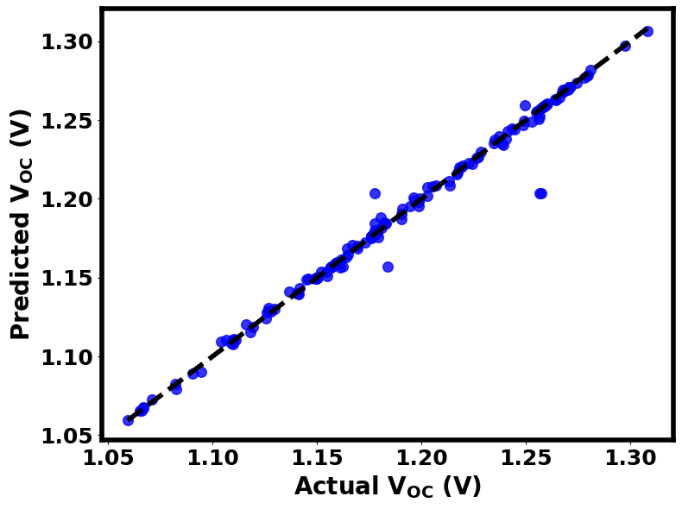** | **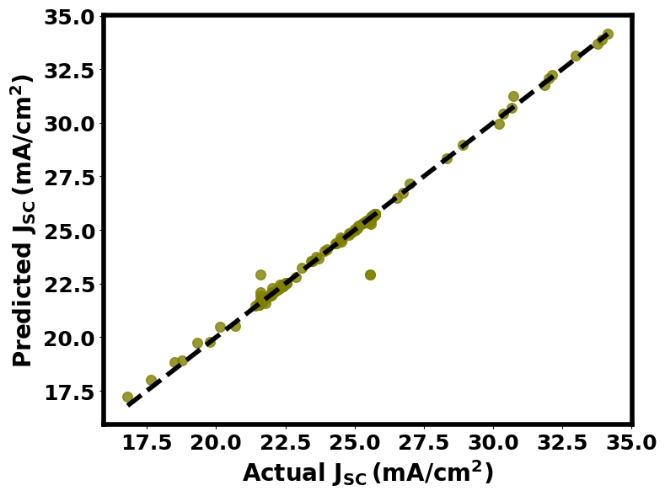** |
| **(c)** | **(d)** |
| **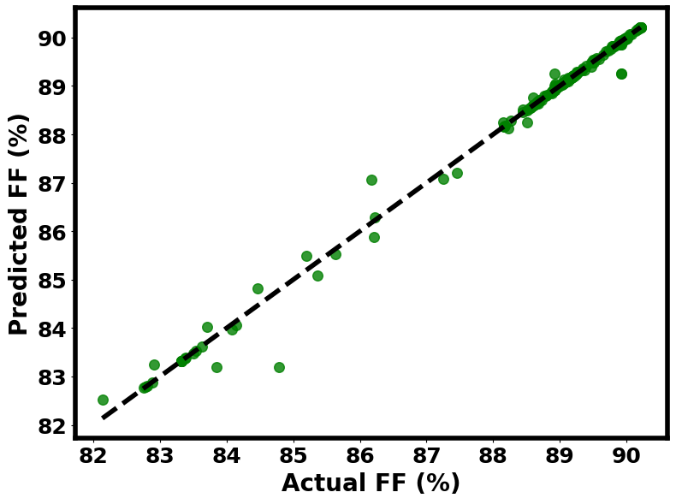** | **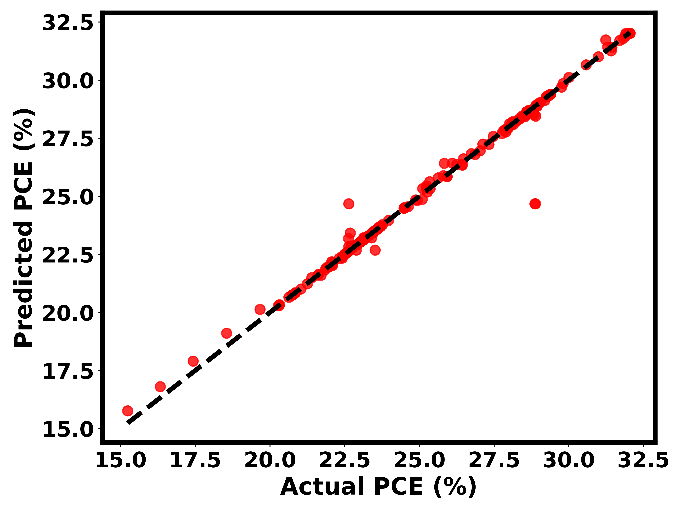** |

**Fig. S5.** Comparison of actual and predicted PV metrics: (a) V_OC_, (b) J_SC_, (c) FF, and (d) PCE.
